# Methane Formation Induced via Face-to-Face Orientation of Cyclic Fe Porphyrin Dimer in Photocatalytic CO_2_ Reduction

**DOI:** 10.3390/molecules29112453

**Published:** 2024-05-23

**Authors:** Yusuke Kuramochi, Masaya Hashimoto, Akiharu Satake

**Affiliations:** 1 Department of Chemistry, Graduate School of Science, Tokyo University of Science, 1-3 Kagurazaka, Shinjuku-ku, Tokyo 162-8621, Japan; 2 Department of Chemistry, Faculty of Science Division II, Tokyo University of Science, 1-3 Kagurazaka, Shinjuku-ku, Tokyo 162-8621, Japan

**Keywords:** carbon dioxide fixation, homogeneous catalysts, iron, photocatalysis, porphyrin

## Abstract

Iron porphyrins are known to provide CH_4_ as an eight-electron reduction product of CO_2_ in a photochemical reaction. However, there are still some aspects of the reaction mechanism that remain unclear. In this study, we synthesized iron porphyrin dimers and carried out the photochemical CO_2_ reduction reactions in *N,N*-dimethylacetamide (DMA) containing a photosensitizer in the presence of 1,3-dimethyl-2-phenyl-2,3-dihydro-1*H*-benzo[d]imidazole (BIH) as an electron donor. We found that, despite a low catalytic turnover number, CH_4_ was produced only when these porphyrins were facing each other. The close proximity of the cyclic dimers, distinguishing them from a linear Fe porphyrin dimer and monomers, induced multi-electron CO_2_ reduction, emphasizing the unique role of their structural arrangement in CH_4_ formation.

## 1. Introduction

**Figure 1:**
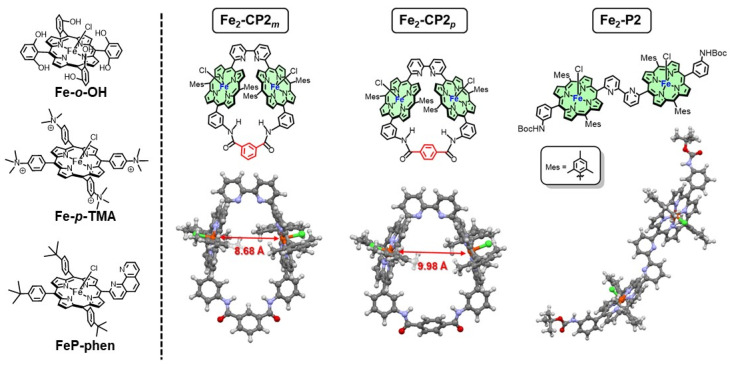
Chemical structures of Fe porphyrins (molecular models optimized by density functional theory calculations using B3LYP/LANL2DZ/6–31G(d) for the Fe porphyrin dimers).

The rise in atmospheric CO_2_ concentration has led to serious impacts on the environment, emphasizing the urgent requirement for CO_2_ reduction. Many attempts have been made to utilize light energy to reduce CO_2_ and convert it into energy-rich substances similar to photosynthesis. One significant challenge is the selective reduction of CO_2_ while suppressing thermodynamically favorable proton reduction, which generates hydrogen, and this has been addressed using metal complexes. For instance, in artificial Z-scheme systems using semiconductor photocatalysts, the incorporation of metal complex catalysts at reduction sites enables selective CO_2_ reduction under visible light irradiation. This process effectively extracts electrons from water, and simultaneously minimizes hydrogen generation, producing CO and formic acid [1,2]. Abundant earth elements have been used as the central metal; among them, Fe porphyrin complexes have been extensively studied owing to their high selectivity and activity [3,4]. The introduction of peripheral proton-donating groups (**Fe-*o*-OH** in Figure 1) considerably improved the catalytic activity of Fe porphyrins, resulting in selective CO production [5]. In addition, linking Fe porphyrins through an *o*-phenylene or a urea group enhanced CO production [6,7]. The Fe porphyrin substituted with *p*-trimethylammoniophenyl groups (**Fe-*p*-TMA** in Figure 1) also exhibited high CO production activity in the electrochemical CO_2_ reduction [8]. Interestingly, under photochemical CO_2_ reduction conditions using tris(2-phenylpyridine)iridium (Ir(ppy)_3_) as a photosensitizer and triethylamine (TEA) as a sacrificial electron donor in acetonitrile, **Fe-*o*-OH**, and **Fe-*p*-TMA** yielded CH_4_ along with CO [9]. The formation of CH_4_ was observed when using an organic photosensitizer, 3,7-(4-biphenyl)-1-naphthalene-10-phenoxazine (Phen2), instead of Ir(ppy)_3_ in *N,N*-dimethylformamide (DMF) solution containing **Fe-*p*-TMA** and sacrificial electron donors [10]. The reaction mechanism indicated the involvement of the Fe(II)–CO intermediate. However, although computational studies have proposed this reaction mechanism [11], the actual formation mechanism of CH_4_, an eight-electron reduction product of CO_2_, remains unclear.

We previously synthesized cyclic Zn porphyrin dimers connected via 2,2′-bipyridine (bpy) and isophthalamide (**Zn_2_-CP2*_m_***) or terephthalamide linkers (**Zn_2_-CP2*_p_***) [12]. Herein, we substituted the central Zn ions of porphyrins with Fe ions to yield two types of cyclic Fe porphyrin dimers with different distances between the porphyrin planes (**Fe_2_-CP2*_m_*** and **Fe_2_-CP2_p_** in Figure 1). The photocatalytic CO_2_ reduction, in the presence of Ir(ppy)_3_ [9] or Phen2 [10] as the photosensitizer and 1,3-dimethyl-2-phenyl-2,3-dihydro-1*H*-benzo[d]imidazole (BIH) [13] as the electron donor, surprisingly produced CH_4_ only when using the cyclic Fe porphyrin dimers (**Fe_2_-CP2*_m_*** and **Fe_2_-CP2_p_**). In contrast, a linear Fe porphyrin dimer (**Fe_2_-P2**) and monomeric Fe porphyrins did not produce CH_4_, indicating that the close proximity of two Fe porphyrins facing each other induced the multielectron reduction of CO_2_.

## 2. Results and Discussion

**Figure 2:**
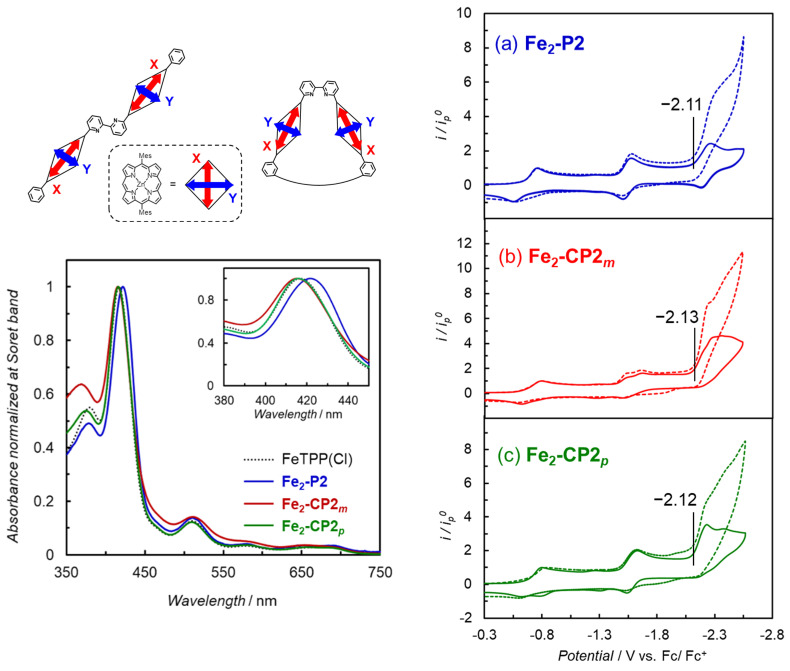
(**Left**) Interaction of transition dipole moments of the bisporphyrin and UV−vis absorption spectra of FeTPP(Cl) (black dotted line; TPP = tetraphenylporphyrin), **Fe_2_-P2** (blue line), **Fe_2_-CP2*_m_*** (red line), **Fe_2_-CP2*_p_*** (green line) in CHCl_3_. The inset shows the magnification of the Soret band region: (**right**) CVs (scan rate = 100 mV s^−1^) of (**a**) **Fe_2_-P2**, (**b**) **Fe_2_-CP2*_m_***, and (**c**) **Fe_2_-CP2*_p_*** (0.3 mM) collected in dry DMF under Ar (solid lines) and CO_2_ (dotted lines) atmospheres with 0.1 M *^n^*Bu_4_NPF_6_ as the supporting electrolyte.

**Fe_2_-CP2*_m_*** and **Fe_2_-CP2_p_** were, respectively, prepared by demetallation of **Zn_2_-CP2*_m_*** and **Zn_2_-CP2_p_ [12]** to obtain the corresponding free-base porphyrins, followed by the introduction of Fe ions. **Fe_2_-P2** was prepared by the introduction of Fe ions into the corresponding free-base precursor [12]. In the UV–vis absorption spectra, the Soret band of **Fe_2_-P2** red shifted compared with those of **Fe_2_-CP2*_m_***, **Fe_2_-CP2_p_**, and Fe(III) tetraphenylporphyrin chloride (FeTPP(Cl)) (Figure 2, left). The red-shifted band is attributed to the head-to-tail excitonic coupling between the two transition dipoles of X in the anticonformation of **Fe_2_-P2**, indicating that a linear structure is stable in **Fe_2_-P2**, as observed for the corresponding Zn porphyrins (Figure 1) [12]. The cyclic voltammogram (CV) and differential pulse voltammogram (DPV) of **Fe_2_-P2** in Ar-saturated DMF showed three reversible redox waves at −0.74, −1.55, and −2.23 V vs. Fc/Fc^+^ (−0.27, −1.08, and −1.76 V vs. SCE) [14], which corresponded, respectively, to Fe(III/II), Fe(II/I), and Fe(I/0) couples (Figure 2, right and Figure S12). The observation of only three redox waves indicates that each porphyrin is reduced independently and that the electronic interaction between the two porphyrins through the bpy linker is negligible. Meanwhile, although the CVs of **Fe_2_-CP2*_m_*** and **Fe_2_-CP2_p_** showed redox waves at the similar positions to those of **Fe_2_-P2**, the second redox wave of **Fe_2_-CP2*_m_*** split into two, indicating that the porphyrins in close proximity, arranged in a face-to-face configuration, exhibit electrical interaction with each other. Under a CO_2_ atmosphere, **Fe_2_-P2**, **Fe_2_-CP2*_m_***, and **Fe_2_-CP2_p_** showed similar catalytic currents at the third waves of Fe(I/0) of the Fe porphyrin dimers (Figure 2, right). Lewis acids, known to enhance the activity of Fe porphyrin catalysts for CO_2_ reduction [15,16], were anticipated to exhibit differences in their interactions with the bpy moiety between cyclic and linear structures. However, no significant difference among three porphyrin dimers in the catalytic currents was observed for the electrochemical CO_2_ reduction even when using water and/or metal ion additives (Figures S13–S15 and Table S1).

We first attempted to perform photocatalytic CO_2_ reduction using **Fe_2_-P2** in the presence of Ir(ppy)_3_ as a photosensitizer and TEA as an electron donor, but no reduction product was detected. The present Fe porphyrin dimers exhibited more negative reduction potentials (−2.17 to −2.23 V vs. Fc/Fc^+^ and −1.70 to −1.76 V vs. SCE for Fe(I/0)) compared with the reported Fe porphyrins, such as **Fe-*o*-OH** (−1.57 V vs. SCE for Fe(I/0)) and **Fe-*p*-TMA** (−1.47 V vs. SCE for Fe(I/0)) [17]. According to the energy diagram (Figure S16), the oxidation quenching process [18] of the excited Ir(ppy)_3_ by either **Fe_2_-P2** or **Fe_2_-CP2*_m_*** is thermodynamically less favorable when using TEA. Consequently, we used BIH with a stronger reducing power as an electron donor instead of TEA. The energy diagram and results of a phosphorescence quenching experiment support that electron transfer from BIH to the excited Ir(ppy)_3_ (i.e., reductive quenching) [18], followed by a reduction in the Fe(I) porphyrins, can occur (Figures S17–S19). Here, BIH was used in much larger quantities (10 mM) than the catalyst (10 μM), and it was expected that the reaction could proceed, although it was slightly unfavorable thermodynamically. Under the reaction conditions for the photocatalytic CO_2_ reduction using BIH and **Fe-*o*-OH**, we observed the production of CO during the catalytic reaction (Table S2). However, the amount of BIH consumed was significantly greater than the production of CO (Figure S21). The NMR spectra after irradiation in the presence of CO_2_ revealed the formation of unidentified BIH decomposition products (Figure S22) rather than the formation of BI^+^, which is typically observed in reactions with the [Ru(bpy)_3_]^2+^ photosensitizer as a two-electron oxidation product of BIH [13]. A highly reactive BI radical, formed via the oxidation and deprotonation of BIH, is likely to react with CO_2_ to generate the unidentified products (Figure S23). This is hypothesized because Ir(ppy)_3_ would not accept the electron from the BI radical. The investigation of the effects of solvents and additives showed that *N,N*-dimethylacetamide (DMA) [19] promoted CO production and suppressed BIH consumption more than acetonitrile (entries 4−6 in Table S3 and Figure S24). Therefore, in the subsequent experiments, the photoreactions were performed using DMA.

**Figure 3:**
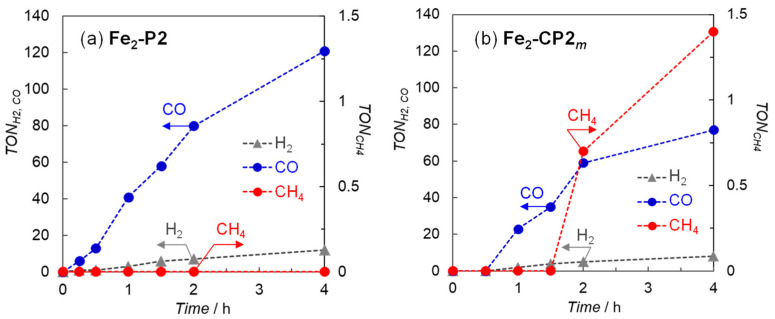
Time dependence of the reduction products generated during the irradiation of CO_2_-saturated DMA solutions (2.0 mL) containing (**a**) **Fe_2_-P2** and (**b**) **Fe_2_-CP2_m_** (10 μM) in the presence of BIH (10 mM) and Ir(ppy)_3_ (0.2 mM) at 450 nm using a merry-go-round apparatus equipped with LED lamps (input power: 10 mW).

Photocatalytic CO_2_ reductions using **Fe_2_-P2** and **Fe_2_-CP2*_m_*** (10 μM) in DMA containing BIH (10 mM) and Ir(ppy)_3_ (0.2 mM) under 450 nm light were performed. The turnover numbers (TONs) of the reduction products against the Fe porphyrin dimers are shown in Figure 3. No detectable amounts of CO, H_2_, or CH_4_ were found in the absence of any one of the Fe porphyrin dimers, Ir(ppy)_3_, and light, whereas only H_2_ was detected under Ar instead of CO_2_. While **Fe_2_-P2** and **Fe_2_-CP2*_m_*** produced CO, the amount of CO was smaller in **Fe_2_-CP2*_m_***. Interestingly, a small amount of CH_4_ was formed in **Fe_2_-CP2*_m_*** (Figure 3b). Under the same conditions, CH_4_ was not detected in **Fe_2_-P2** (Figure 3a), **Fe-*o*-OH**, or **FeP-phen**, which is a model monomer with a diimine ligand. Figure 3b shows CH_4_ production with an induction period and lower CO production than that in Figure 3a, indicating that the CH_4_ was formed via the reduction in CO, as reported previously [9,10].

**Figure 4:**
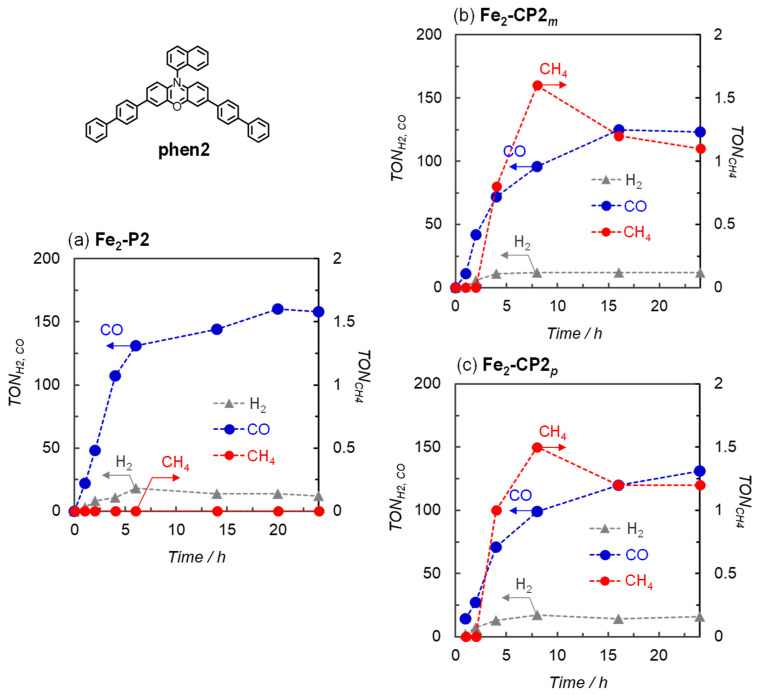
Time dependence of the reduction products generated during the irradiation of CO_2_-saturated DMA solutions (2.0 mL) containing (**a**) **Fe_2_-P2**, (**b**) **Fe_2_-CP2_m_**, and (**c**) **Fe_2_-CP2_p_** (10 μM) in the presence of BIH (10 mM) and Phen2 (1 mM) at 420 nm using a merry-go-round apparatus equipped with LED lamps (Input power: 5 mW).

Next, we investigated the photocatalytic CO_2_ reduction reactions in which the organic dye Phen2, rather than Ir(ppy)_3_, was used as the photosensitizer (Figure 4) [10]. During the irradiation of the solution in the absence of the catalyst (Figure S28), BIH decomposition was still observed without reduction products of CO_2_. However, reducing the light intensity to 5 mW suppressed BIH decomposition. When **Fe_2_-P2** was used as the catalyst, a linear CO formation was observed for up to 4 h under the 5 mW light intensity (Figure S29). Figure 4 shows the TONs of the reduction products against the Fe porphyrin dimers in DMA containing BIH (10 mM) and Phen2 (1.0 mM) under 420 nm light (5 mW). As observed with Ir(ppy)_3_, CH_4_ was formed with induction periods in the cyclic structure (**Fe_2_-CP2*_m_*** and **Fe_2_-CP2_p_**), whereas no CH_4_ was detected in the linear structure (**Fe_2_-P2**). In addition, the CO productions in Figure 4b,c were smaller than those in Figure 4a. In the previous systems involving **Fe-*p*-TMA** as the catalyst and TEA as the electron donor, the addition of a proton source, such as trifluoroethanol (TFE), enhanced the formation of CO and CH_4_ [9,10]. However, using the cyclic porphyrin dimer, the CH_4_ production decreased with the addition of TFE and was completely suppressed by PhOH, while TFE and PhOH enhanced the CO production. The addition of Mg ions decreased the production of CO and CH_4_ (Figure S30).

**Figure 5:**
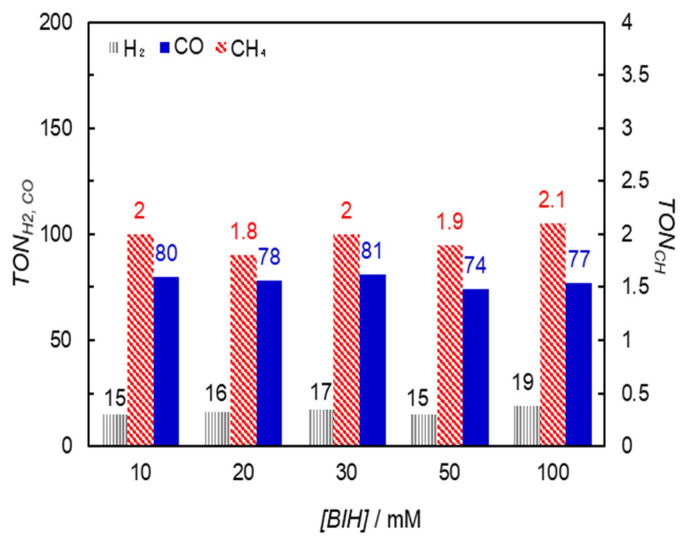
Relationship between the reduction products and the initial concentration of BIH during irradiation at 420 nm for 4 h in CO_2_-saturated DMA solutions (2.0 mL) containing **Fe_2_-CP2*_p_*** (10 μM) and Phen2 (1 mM), using a merry-go-round apparatus equipped with LED lamps (input power: 5 mW).

In conventional catalytic reactions using Phen2 as the photosensitizer, the reaction typically involves an oxidative quenching process, where electrons are transferred from the excited Phen2 to the catalyst [18,20]. However, this system would proceed via a reductive quenching process involving electron transfer from the electron donor to the excited Phen2. The fluorescence quenching experiments of Phen2 by BIH demonstrate that the electron transfer from BIH to the excited singlet state of Phen was efficient (Figure S32). However, the quenching efficiency of the excited Phen2 (*η_q_*), which was estimated from the Stern–Volmer plot [18], strongly depended on the concentration of BIH because of the shorter fluorescence lifetime ([BIH] = 10 mM, *η_q_* = 7%; [BIH] = 100 mM, and *η_q_* = 41%). Meanwhile, we observed that the TONs were less dependent on the BIH concentration (10−100 mM in Figure 5), suggesting that the electron transfer from BIH mainly occurred not via the excited singlet state but via the long-lived excited triplet state of Phen2 [21], which has a lifetime of 480 μs [22].

The photocatalytic CO_2_ reduction using **Fe_2_-CP2*_p_*** and 100 mM BIH produced CH_4_ with TON = 3.5 against the Fe porphyrin dimer after 35 h, exceeding the amount of the Fe porphyrin units (Figure S34). The capillary electrophoresis showed that the TON of formic acid reached 38 after irradiation for 18 h in the presence of **Fe_2_-CP2*_p_*** and BIH (100 mM, Figure S34). We conducted isotopic experiments under ^12^CO_2_ and ^13^CO_2_ atmospheres. In gas chromatography/mass spectrometry, ^13^CO (*m*/*z* = 29) and ^13^CH_4_ (*m*/*z* = 17) were detected under a ^13^CO_2_ atmosphere (Figures S35 and S36), confirming that the carbon source of CO and CH_4_ was CO_2_. The ^1^H and ^13^C NMR spectra were measured in a DMA-*d*_9_ solution during irradiation under ^12^CO_2_ and ^13^CO_2_ atmospheres (Figures S37–S39). The spectral changes during light irradiation showed that BIH was almost completely consumed after 21 h, indicating that the catalytic reaction stopped due to the disappearance of BIH. No reduction products, including methanol or formaldehyde, were observed, except for formic acid, which showed a doublet peak at 8.68 ppm with a coupling constant of *J*_13C-H_ = 175 Hz and a singlet peak at 8.72 ppm in ^1^H NMR spectra under ^12^CO_2_ and ^13^CO_2_ atmospheres, respectively (Figure S38). A peak at 167 ppm was assigned to HC(O)-, which was correlated with the doublet proton peak at 8.68 ppm in the heteronuclear multiple bond connectivity (HMBC), observed in the ^13^C NMR spectrum under only a ^13^CO_2_ atmosphere (Figures S39 and S40) [19]. Formic acid was not observed in the absence of the Fe porphyrins, indicating that formic acid is produced via the CO_2_ reduction catalyzed by the Fe porphyrins, and it is not directly formed by the chemical reaction between BIH and CO_2_ [23,24]. Furthermore, a peak appeared at 222 ppm in the ^13^C NMR spectrum only under a ^13^CO_2_ atmosphere (indicated by an asterisk in Figure S39). The peak can be attributed to the Fe–^13^CO signal [25], indicating the formation of a carbonyl intermediate during irradiation, as observed in previous reports. In addition, only under a ^13^CO_2_ atmosphere an intense peak was observed at 172 ppm (Figure S39), which was correlated with the proton peaks at 7.4, 3.4, 2.7, 2.4, and 1.8 ppm in HMBC (Figure S40). Although no clear attribution could be established, it likely corresponded to the reaction products of BIH and CO_2_, an adduct of CO_2_ with the BI radical caused by the oxidation and deprotonation of BIH.

## 3. Materials and Methods

### 3.1. General Procedure

All chemicals and solvents were of commercial reagent quality and were used without further purification unless otherwise stated. Tris(2-phenylpyridine)iridium (Ir(ppy)_3_) was purchased from Sigma-Aldrich (St. Louis, MO, USA). Fe(III) 5,10,15,20-tetrakis(2,6-dihydroxyphenyl)porphyrin chloride (**Fe-*o*-OH**) [5], 3,7-(4-biphenyl)-1-naphthalene-10-phenoxazine (Phen2) [10], the cyclic Zn porphyrin dimers (**Zn_2_-CP2*_m_*** and **Zn_2_-CP2*_p_***), the linear free-base porphyrin dimer (**Fb_2_-P2**) [12], 1,3-dimethyl-2-phenyl-2,3-dihydro-1*H*-benzo[d]imidazole (BIH) [13], and 5,10,15-tris(4-*tert*-butylphenyl)-20-(1,10-phenanthrolin-2-yl)-21*H*,23*H*-porphyrin (**H_2_P-phen**) [26] were prepared according to the literature. *N,N*-dimethylformamide (DMF) was dried over molecular sieves of size 4 Å. The reactions were monitored on silica gel 60F_254_ TLC plates (Merck, Darmstadt, Germany). The following silica gels utilized for the column chromatography were purchased from Kanto Chemical Co. Inc. (Tokyo, Japan): silica gels (Spherical, Neutral) 40–100 μm and (Flash) 40–50 μm. ^1^H, ^13^C NMR, and ^1^H-^1^H correlation spectroscopy (COSY), ^1^H−^13^C heteronuclear single quantum correlation (HSQC), and ^1^H−^13^C heteronuclear multiple bond correlation (HMBC) spectra were recorded using a JEOL JNM-ECZ-400 and a JEOL JNM-ECA-500 (JEOL, Tokyo, Japan). Chemical shifts were recorded in parts per million (ppm) relative to tetramethylsilane. MALDI–TOF mass spectra were collected on a JEOL JMS S-3000 with dithranol as a matrix with sodium iodide (NaI). UV-vis absorption spectra were collected using a square cell (path length = 1.0 cm) on a JASCO V-650 spectrometer (JASCO, Tokyo, Japan). The steady-state emission spectra were collected on an Hitachi F-4500 spectrometer and corrected for the response of the detector system. The fluorescence intensities were normalized at the absorbance of the excitation wavelength. Cyclic voltammogram (CV) and differential pulse voltammogram (DPV) were measured using an ALS-H/CHI Model 612E electrochemical analyzer (BAS, Tokyo, Japan) in a micro-cell equipped with a glassy carbon working electrode (*ϕ* 1.6 mm) and a Pt counter electrode. The micro-cell was connected via a Luggin capillary with a reference electrode of Ag/AgNO_3_ (10 mM in DMA). Tetrabutylammonium hexafluorophosphate (*^n^*Bu_4_NPF_6_) recrystallized from ethyl acetate was used as a supporting electrolyte. Ferrocene was used as an external standard, and all potentials were referenced to the ferrocene/ferrocenium couple. The currents (*i*) were normalized by the peak currents (*i_p_^0^*) corresponding to the Fe(III)/Fe(II) wave in the absence of CO_2_ and additives. High-performance liquid chromatographies (HPLCs) were carried out using a JASCO PU-2089 and an MD-44010 system (JASCO, Tokyo, Japan) equipped with a TSKgel ODS-100S column (4.6 mm I.D. × 25 cm; Tosoh, Tokyo, Japan) using acetonitrile/H_2_O = 4/1 (*v*/*v*) as an eluent.

### 3.2. Synthesis of Fb_2_-CP2_m_

The TFA (0.50 mL) was slowly added to **Zn_2_-CP2*_m_*** (10 mg, 5.9 × 10^−6^ mol) in a 10 mL flask, and the mixture was stirred for 2 h. The solution was slowly poured into a saturated NaHCO_3_ aqueous solution in an ice bath. The organic layer was transferred to a PFA-coated funnel, and the aqueous layer was extracted with CHCl_3_ (10 mL × 3). The combined organic layer was washed with water and dried over anhydrous Na_2_SO_4_. The solvent was evaporated to dryness, resulting in a purple solid, **Fb_2_-CP2*_m_*** (8.3 mg, 90%). ^1^H NMR (500 MHz, pyridine-*d*_5_) δ/ppm = 10.85 (s, 2H, NH), 8.91 (s, 1H, Ph), 8.83 (m, 2H, Ph), 8.74 (m, 4H, *β*-pyrrole), 8.70 (m, 2H, Py), 8.58–8.47 (m, 12H, *β*-pyrrole, Ph, Py), 8.36 (m, 4H, *β*-pyrrole), 8.31 (m, 2H, Py), 8.11 (m, 2H, Ph), 7.74 (s, 2H, Ph), 7.67 (m, 2H, Ph), 7.55 (m, 1H, Ph), 7.27 (s, 4H, mesityl), 7.08 (s, 4H, mesityl), 2.48 (s, 12H, CH_3_), 1.64 (s, 12H, CH_3_), 1.54 (s, 12H, CH_3_), −2.84 (s, 4H, inner NH); MALDI-TOF mass: *m*/*z* [M + H]^+^ 1557.6808, calcd. for [C_106_H_85_N_12_O_2_]^+^ 1557.6913.

### 3.3. Synthesis of Fe_2_-CP2_m_

In a 10 mL flask were placed **Fb_2_-CP2*_m_*** (8.3 mg, 5.3 × 10^−6^ mol), CHCl_3_ (2.0 mL), and 2,6-lutidine (15 μL, 1.3 × 10^−4^ mol). Anhydrous FeCl_2_ (37 mg, 2.9 × 10^−4^ mol) dissolved in methanol (1.0 mL) was added to it, and the mixture was stirred under reflux for 18 h. The resulting solution was transferred to a perfluoroalkoxy alkane (PFA)-coated flask, and diluted with CHCl_3_ (ca. 10 mL). The organic solution was washed with 1 M HCl aqueous solution (×3) and water (×3) and then dried over anhydrous Na_2_SO_4_. The residue obtained by evaporation of the solvent was purified with a flush silica gel column (eluents: CHCl_3_, CHCl_3_:CH_3_OH = 50:1, and CHCl_3_:CH_3_OH = 10:1). The fraction eluted with CHCl_3_:CH_3_OH = 50:1 was collected and the solvent was evaporated. The solid was dissolved in CHCl_3_ (ca. 10 mL) was treated with 1 M HCl aqueous solution (×3) and water (×3), and passed through Phase Separator paper (Whatman, Maidstone, UK). The solvent was evaporated to dryness, giving the titled compound as a black solid (7.9 mg, 86%). MALDI-TOF mass: *m*/*z* [M − 2Cl + H]^+^ 1666.5287 (max), calcd. for [C_106_H_81_N_12_O_2_Fe_2_]^+^ 1666.5333; UV-vis absorption (CHCl_3_) *λ*_max_/nm (ε/M^−1^cm^−1^) = 373 (6.2 × 10^4^, LMCT band), 415 (1.1 × 10^5^, Soret band), and 509 (1.4 × 10^4^, Q band).

### 3.4. Synthesis of Fb_2_-CP2_p_

The TFA (1.0 mL) was slowly added to **Zn_2_-CP2*_p_*** (8.7 mg, 5.2 × 10^−6^ mol) in a 10 mL flask, and the mixture was stirred for 2 h. The solution was slowly poured into a saturated NaHCO_3_ aqueous solution in an ice bath. The organic layer was transferred to a PFA-coated funnel and the aqueous layer was extracted with CHCl_3_ (10 mL × 3). The combined organic layer was washed with water and dried over anhydrous Na_2_SO_4_. The solvent was evaporated to dryness, giving a purple solid, **Fb_2_-CP2*_p_*** (7.1 mg, 89%). ^1^H NMR (400 MHz, CDCl_3_) δ/ppm = 8.67 (d, *J* = 8.2 Hz, 2H, Ph or Py), 8.63 (d, *J* = 7.8 Hz, 2H, Ph or Py), 8.52 (d, *J* = 4.7 Hz, 4H, *β*-pyrrole), 8.47 (t, *J* = 7.8 Hz, 2H, Ph or Py), 8.43–8.45 (2H, Ph or Py), 8.42 (d, J = 4.7 Hz, 4H, *β*-pyrrole), 8.39 (d, *J* = 4.7 Hz, 4H, *β*-pyrrole), 8.35 (d, *J* = 4.7 Hz, 4H, *β*-pyrrole), 8.11 (d, *J* = 7.3 Hz, 2H, Ph or Py), 7.70 (t, *J* = 7.9 Hz, 2H, Ph or Py), 7.59 (s, 4H, Ph), 7.19 (s, 4H, mesityl), 7.13 (s, 4H, mesityl), 7.00 (brs, 2H, Ph or Py), 2.59 (s, 12H, CH_3_), 1.48 (s, 12H, CH_3_), 1.36 (s, 12H, CH_3_), −3.44 (s, 4H, inner NH); MALDI-TOF mass: *m*/*z* [M + H]^+^ 1557.6928, calcd. for [C_106_H_85_N_12_O_2_]^+^ 1557.6913.

### 3.5. Synthesis of Fe_2_-CP2_p_

In a 10 mL flask were placed **Fb_2_-CP2*_p_*** (7.1 mg, 4.6 × 10^−6^ mol), CHCl_3_ (2.0 mL), and 2,6-lutidine (14 μL, 1.2 × 10^−4^ mol). Anhydrous FeCl_2_ (37 mg, 2.9 × 10^−4^ mol) dissolved in methanol (1.0 mL) was added to it, and the mixture was stirred under reflux for 18 h. The resulting solution was transferred to a perfluoroalkoxy alkanes (PFA)-coated flask, and diluted with CHCl_3_ (ca. 10 mL). The organic solution was washed with 1 M HCl aqueous solution (×3) and water (×3), and then dried over anhydrous Na_2_SO_4_. The residue obtained by evaporation of the solvent was purified with a flush silica gel column (eluents: CHCl_3_, CHCl_3_:CH_3_OH = 50:1, and CHCl_3_:CH_3_OH = 10:1). The fraction eluted with CHCl_3_:CH_3_OH = 50:1 was collected and the solvent was evaporated. The solid was dissolved in CHCl_3_ (ca. 10 mL) was treated with 1 M HCl aqueous solution (×3) and water (×3) and passed through Phase Separator paper (Whatman). The solvent was evaporated to dryness, resulting in the titled compound as a black solid (6.3 mg, 79%). MALDI-TOF mass: *m*/*z* [M−2Cl + H]^+^ 1666.5417 (max), calcd. for [C_106_H_81_N_12_O_2_Fe_2_]^+^ 1666.5333; UV-vis absorption (CHCl_3_) *λ*_max_/nm (ε/M^−1^cm^−1^) = 375 (7.3 × 10^4^, LMCT band), 416 (1.7 × 10^5^, Soret band), 510 (1.4 × 10^4^, Q band).

### 3.6. Synthesis of Fe_2_-P2

In a 10 mL flask were placed **5Fb_2_** (26 mg, 1.6×10^−5^ mol), CHCl_3_ (5.0 mL), and 2,6-lutidine (30 μL, 2.5 × 10^−4^ mol). Anhydrous FeCl_2_ (100 mg, 7.9 × 10^−4^ mol) dissolved in methanol (3.0 mL) was added to it, and the mixture was stirred under reflux for 24 h. The reaction was quenched by adding EDTA aqueous solution, and the resulting solution was transferred to a perfluoroalkoxy alkanes (PFA)-coated flask, and diluted with CHCl_3_ (ca. 50 mL). The organic solution was washed with water (×3) and brine (×1), and then dried over anhydrous Na_2_SO_4_. The use of hydrochloric acid was avoided because the Boc group could be removed under acidic conditions. The residue obtained by evaporation of the solvent was purified with a flush silica gel column (eluents: CHCl_3_:CH_3_OH = 50:1 and CHCl_3_:CH_3_OH = 10:1). The fraction eluted with CHCl_3_:CH_3_OH = 10:1 was collected, and was stirred with brine for overnight. The organic layer was passed through Phase Separator paper (Whatman) and the solvent was evaporated to dryness, giving the titled compound as a black solid (24 mg, 85%). MALDI-TOF mass: *m*/*z* [M − 2Boc − 2Cl + H]^+^ 1534.5110 (max), calcd. for [C_98_H_76_Fe_2_N_12_]^+^ 1534.5121; UV-vis absorption (CHCl_3_) *λ*_max_/nm (ε/M^−1^cm^−1^) = 378 (8.4 × 10^4^, LMCT band), 421 (1.8 × 10^5^, Soret band), 509 (2.4 × 10^4^, Q band).

### 3.7. Synthesis of FeP-phen

In a 25 mL flask were placed **H_2_P-phen** (30 mg, 3.4 × 10^−5^ mol), CHCl_3_ (8.0 mL), and 2,6-lutidine (35 μL, 3.0 × 10^−4^ mol). Anhydrous FeCl_2_ (107 mg, 8.5 × 10^−4^ mol) dissolved in methanol (3.0 mL) was added to it, and the mixture was stirred under reflux for 22 h. The reaction was quenched by adding EDTA (ethylenediaminetetraacetic acid) aqueous solution, and the resulting solution was transferred to a perfluoroalkoxy alkanes (PFA)-coated flask, and diluted with CHCl_3_ (ca. 50 mL). The organic solution was washed with was washed with 1 M HCl (×3) and brine and then dried over anhydrous Na_2_SO_4_. The residue obtained by evaporation of the solvent was purified with a flush silica gel column (eluents: CHCl_3_, CHCl_3_:CH_3_OH = 9:1). The fraction eluted with CHCl_3_:CH_3_OH = 9:1 was collected and the solvent was evaporated. The solid was dissolved in CHCl_3_ (ca. 10 mL) and treated with 1 M HCl aqueous solution (×3) and brine, and passed through Phase Separator paper (Whatman). The solvent was evaporated to dryness, giving the titled compound as a black solid (22 mg, 69%). MALDI-TOF mass: *m*/*z* [M − Cl + H]^+^ 939.3940 (max), calcd. for [C_62_H_55_FeN_6_]^+^ 939.3833.

### 3.8. Photocatalytic CO_2_ Reduction

In glass tubes (8.0 mL, i.d. = 10 mm), 1.0 mL of CO_2_-saturated DMA solutions containing BIH was added by 1.0 mL of Ar-saturated DMA solutions containing the Fe porphyrin dimer and the photosensitizer, and the reaction solutions were bubbled with CO_2_ gas (purity ≥ 99.995%) for 15 min. Photo-irradiations were carried out using a merry-go-round irradiation apparatus (Iris-MG, Cell Systems, Yokohama, Japan) equipped with LED lamps at λ = 420 nm (FWHM = 18.4 nm). The gaseous reaction products (CO, H_2_, and CH_4_) were quantified with a gas chromatography system (GC-2014, Shimadzu Science, Kyoto, Japan) equipped with a Shincarbon column (i.d. 3.0 mm × 3.0 m) and a thermal conductivity detector (TCD). The product (formate) in the solutions was analyzed with a capillary electrophoresis system (Otuka Electronics Co. CAPI-3300I, Osaka, Japan).

### 3.9. 13CO_2_-Labeling Experiment

In glass tubes (8.0 mL, i.d. = 10 mm), 2.0 mL of Ar-saturated DMA solutions containing BIH (0.10 M) was added by 2.0 mL of Ar-saturated DMA solutions containing **Zn_2_-CP2*_p_*** (10 μM) and Phen2 (1.0 mM), and the reaction solutions were bubbled with ^13^CO_2_ gas, which was generated by the addition of 3.0 M sulfuric acid (5 mL) to Ba^13^CO_3_ powder (2.5 g). After irradiation at 420 nm (5 mW) for 16 h with the merry-go-round irradiation apparatus, the gaseous reaction products were analyzed with a GC-MS (GCMS-QP2010 Plus, Shimadzu Science; RESTEK (Bellefonte, PA, USA); RT-Msieve 5A). In an NMR tube, a 0.5 mL DMA-*d*_9_ solution containing **Zn_2_-CP2*_p_*** (0.10 mM), Phen2 (1.0 mM) and BIH (0.10 M) was bubbled with ^13^CO_2_ gas, which was generated by addition of 3.0 M sulfuric acid (3 mL) to Ba^13^CO_3_ powder (1.0 g). After irradiation at 420 nm (5 mW) for 21 h with the merry-go-round irradiation apparatus, the ^1^H and ^13^C NMR spectra were measured.

### 3.10. Computational Methods

The DFT calculations were carried out using the Gaussian 09 package of programs [27]. Each structure was fully optimized using the B3LYP functional using the 6–31G(d) basis set for all atoms, except Fe, and the standard double-ζ type LANL2DZ basis set with the effective core potential of Hay−Wadt for Fe. The stationary points were verified using the vibrational analysis.

## 4. Conclusions

In this study, we show that the photochemical CO_2_ reduction yielded CH_4_, an eight-electron reduction product of CO_2_, when Fe porphyrins were placed in a face-to-face arrangement. Although the catalytic turnover number of CH_4_ was low in this study, this can be attributed to the significant degradation of BIH. Thus, it is expected that by addressing this issue, we can enhance the catalytic performance. While bimetallic porphyrin complexes have been reported to enhance CO production, to the best of our knowledge, this is the first report on the induction of CH_4_ production using bimetallic porphyrins. We anticipate that this finding can contribute to the understanding CH_4_ formation mechanisms and provide potential molecular design guidelines for selective CH_4_ generation.

## Data Availability

The original contributions presented in the study are included in the article/Supplementary Materials, and further inquiries can be directed to the corresponding author/s.

## Supplementary Materials

The following supporting information can be downloaded at: https://www.mdpi.com/article/10.3390/molecules29112453/s1, Figure S1: UV–vis absorption and fluorescence spectra of **Zn_2_-CP2*_m_*** and **Fb_2_-CP2***_m_*; Figure S2: MALDI-TOF mass of **Fb_2_-CP2*_m_***; Figure S3: ^1^H NMR spectrum of **Fb_2_-CP2*_m_***; Figure S4: UV–vis absorption and fluorescence spectra of **Fb_2_-CP2*_m_*** and HCl-treated **Fe_2_-CP2***_m_*; Figures S5–S6: MALDI-TOF mass of **Fe_2_-CP2*_m_*** and **Fb_2_-CP2*_p_***; Figure S7: ^1^H NMR spectrum of **Fb_2_-CP2**; Figure S8: UV–vis absorption spectra of **Fe_2_-CP2*_p_*** before and after treatment with HCl aqueous solution; Figure S9: MALDI-TOF mass of **Fe_2_-CP2*_p_***; Figure S10: UV-vis absorption and fluorescence spectra of **Fb_2_-P2** and brine-treated **Fe_2_-P2**; Figure S11: MALDI-TOF mass of **Fe_2_-P2**; Figure S12: DPVs of **Fe_2_-P2**, **Fe_2_-CP2*_m_***, and **Fe_2_-CP2*_p_***; Figure S13: CVs of **Fe_2_-P2**, **Fe_2_-CP2*_m_***, and **Fe_2_-CP2*_p_*** collected in CO_2_-saturated DMF in the presence of water; Figure S14: CVs of **Fe_2_-P2** and **Fe_2_-CP2*_m_*** collected in CO_2_-saturated DMF in the presence of M(OTf)_n_ (M = Mg (*n* =2), La and Gd (*n* = 3)); Figure S15: CVs of **Fe_2_-P2** and **Fe_2_-CP2*_m_*** in CO_2_-saturated DMF in the presence of M(OTf)_n_ (M = Mg (*n* =2), La and Gd (*n* = 3)), and water; Table S1: Effect of each additive on the ratio of the maximum current to *i_p_^0^*; Figure S16: Energy diagram of the photoinduced electron transfer during the formation of Fe(0) species via the oxidative quenching process by either **Fe-*o*-OH** or the Fe porphyrin dimer, when Ir(ppy)_3_ is used as a photosensitizer and TEA as a sacrificial donor; Figure S17: Energy diagram of the photoinduced electron transfer during the formation of Fe(0) species via the reductive quenching process by BIH, when Ir(ppy)_3_ is used as a photosensitizer and BIH as a sacrificial donor; Figure S18: UV–vis absorption and emission spectra of Ir(ppy)_3_ in Ar-saturated DMA at 298 K in the presence of various amounts of BIH; Figure S19: Stern–Volmer plot of emission quenching of Ir(ppy)_3_ by BIH; Table S2: Photocatalytic reaction using **Fe-*o*-OH** in acetonitrile; Figure S20: HPLC charts of the reaction solutions containing BIH and Ir(ppy)_3_ before and after irradiation; Figure S21: Comparison of the reduction products and BIH consumption in Table S2; Figure S22: ^1^H NMR spectra of the reaction solutions containing BIH and Ir(ppy)_3_ before and after irradiation; Figure S23: Decomposition of BIH during irradiation in the presence of PS and CO_2_; Table S3: Effects of solvents and additives on photocatalytic reaction using **Fe-*o*-OH**; Figure S24: Comparison of the reduction products and BIH consumption in Table S3; Figure S25: Plots of the amounts of the reduction products and the consumption of BIH after 60 min of irradiation at 450 nm using a merry-go-round apparatus equipped with LED lamps versus the concentration of **FeP-phen** in CO_2_-saturated DMA in the presence of Ir(ppy)_3_ and BIH; Figure S26: Time dependence of the reduction products during the irradiation of CO_2_-saturated DMA solutions containing **FeP-phen** in the presence of BIH and Ir(ppy)_3_ at 450 nm; Figure S27: HPLC charts of the resulting solutions after irradiation in Figure S26, and time dependence of the remaining amount of BIH determined using HPLC; Figure S28: Time dependence of the remaining amount of BIH as per irradiation intensity in blank CO_2_-saturated DMA solutions containing BIH and Phen2 during irradiation at 420 nm; Figure S29: Dependence of CO production on light intensity at 420 nm in CO_2_-saturated DMA solutions containing **Fe_2_-P2**, BIH, and Phen2; Figure S30: TONs of CO, H_2_, and CH_4_ during irradiation at 420 nm for 18 h in CO_2_-saturated DMA solutions containing **Fe_2_-CP2*_p_***, BIH, and Phen2 in the presence of acids; Figure S31: UV–vis absorption and fluorescence spectra of Phen2 in Ar-saturated DMA in the presence of various amounts of BIH; Figure S32: Stern–Volmer plot of emission quenching of Phen2 by BIH, Figure S33: Relationship between the reduction products and the initial concentration of BIH during irradiation at 420 nm for 4 h and 18 h in CO_2_-saturated DMA solutions containing **Fe_2_-CP2*_p_*** and Phen2; Figure S34: Time dependence of the reduction products during the irradiation of CO_2_-saturated DMA solutions containing **Fe_2_-CP2*_p_*** in the presence of BIH and Phen2 at 420 nm, and gas chromatogram of the gaseous reaction products after the irradiation for 35 h; Figure S35: Gas chromatograms of the resulting gas-phase products after irradiation at 420 nm for 14 h under ^12^CO_2_ or ^13^CO_2_ atmosphere in DMA solutions containing **Fe_2_-CP2*_p_***, BIH, and Phen2 obtained using mass spectroscopy; Figure S36: Mass spectra of CH_4_ generated under ^12^CO_2_ and ^13^CO_2_ atmosphere, and gas chromatogram of the products obtained from the reaction under ^13^CO_2_ atmosphere, plotted for each *m*/*z*; Figure S37: ^1^H NMR spectra of the reaction solutions containing **Fe_2_-CP2*_p_***, BIH, and Phen2 during irradiation at 420 nm under a ^13^CO_2_ atmosphere; Figure S38: Comparison of the ^1^H NMR spectra of reaction solutions containing **Fe_2_-CP2*_p_***, BIH, and Phen2 after irradiation at 420 nm for 21 h under ^12^CO_2_ and ^13^CO_2_ atmospheres; Figure S39: ^13^C NMR spectra of the reaction solutions containing **Fe_2_-CP2*_p_***, BIH, and Phen2 during irradiation at 420 nm under a ^13^CO_2_ atmosphere; Figure S40: HMBC of the reaction solutions containing **Fe_2_-CP2*_p_***, BIH, and Phen2 during irradiation for 21 h at 420 nm under ^12^CO_2_ and ^13^CO_2_ atmospheres; Figure S41: HSQC of the reaction solutions containing **Fe_2_-CP2*_p_***, BIH, and Phen2 during irradiation for 21 h at 420 nm under a ^13^CO_2_ atmosphere; Figure S42: ^1^H-^1^H COSY of the reaction solutions containing **Fe_2_-CP2*_p_***, BIH, and Phen2 during irradiation for 21 h at 420 nm under a ^13^CO_2_ atmosphere. Scheme S1. Synthetic routes of **Fe2-CP2*m, p*.** Scheme S2. Synthetic route of **Fe2-P2**. Scheme S3. Synthetic route of **FeP-phen**.
