# Supplementary material for: Methane Formation Induced via Face-to-Face Orientation of Cyclic Fe Porphyrin Dimer in Photocatalytic CO2 Reduction
Source: Molecules. 2024 May 23;29(11):2453. doi: 10.3390/molecules29112453 (PMC11174001; doi:10.3390/molecules29112453)
Supplement: Supplementary file 1 [file molecules-29-02453-s001.zip › molecules-3000155-supplementary.pdf]

Supplementary Materials

Methane Formation Induced via Face-to-Face Orientation of Cyclic Fe Porphyrin Dimer in Photocatalytic CO_2 Reduction

Yusuke Kuramochi [1,2,3,*], Masaya Hashimoto [1], Akiharu Satake [1,2,*]

1 Department of Chemistry, Graduate School of Science, Tokyo University of Science, 1-3 Kagurazaka, Shinjuku-ku, Tokyo 162-8621, Japan

2 Department of Chemistry, Faculty of Science Division II, Tokyo University of Science, 1-3 Kagurazaka, Shinjuku-ku, Tokyo 162-8621, Japan

* Correspondence: kuramo@iis.u-tokyo.ac.jp (Y.K.); asatake@rs.tus.ac.jp (A.S.)

† Current address: Institute of Industrial Science, The University of Tokyo, 4-6-1 Komaba, Meguro-ku, Tokyo 153-8505, Japan.

Scheme S1. Synthetic routes of **Fe$_2$-CP2*$_{m,\,p}$***

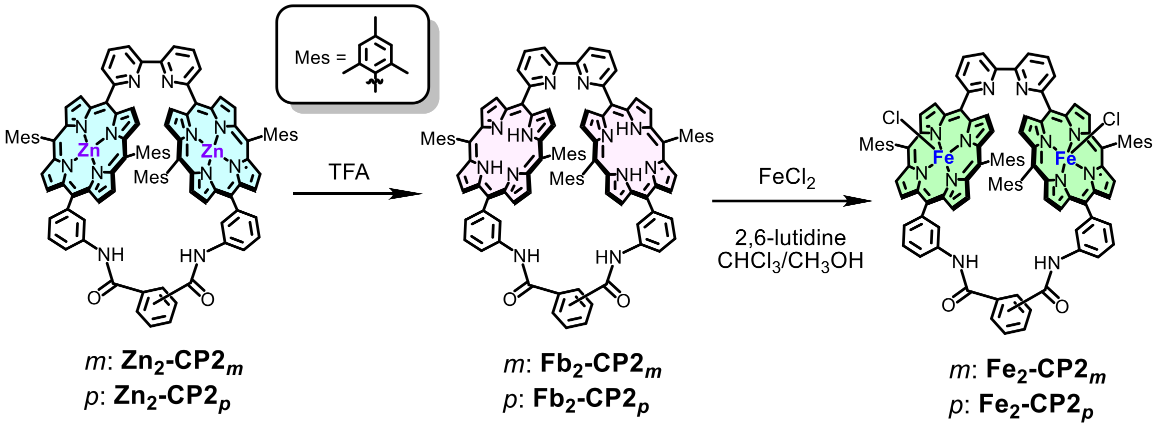

Scheme S2. Synthetic route of **Fe$_2$-P2**.

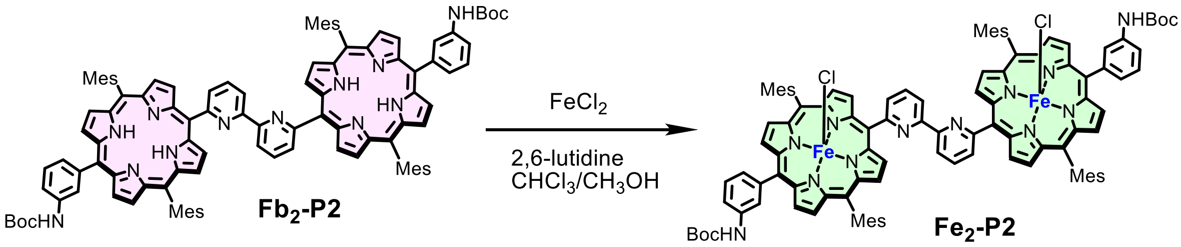

Scheme S3. Synthetic route of **FeP-phen**.

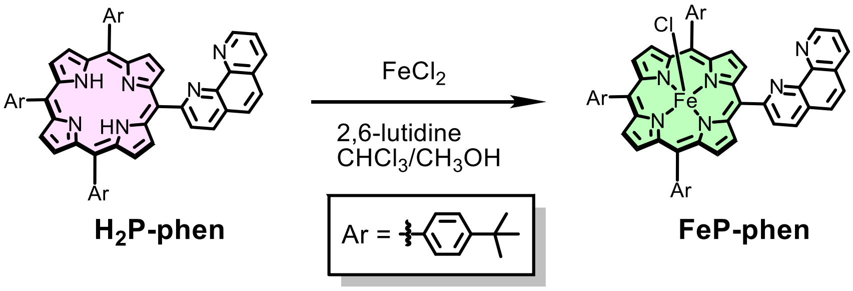

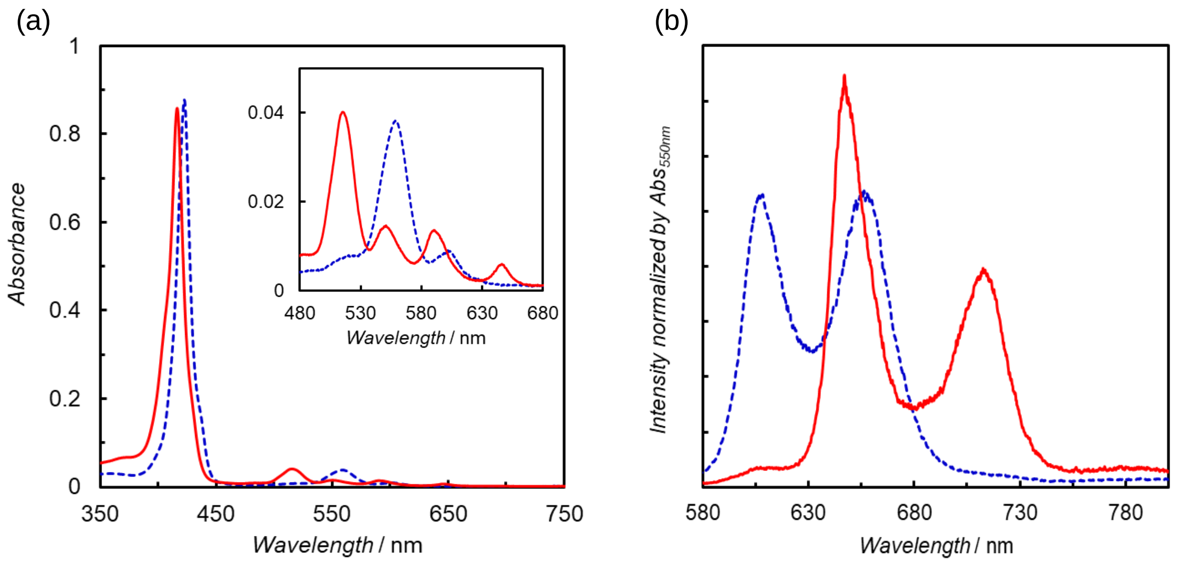

Figure S1. (a) UV–vis absorption spectra and (b) fluorescence spectra (λ_{ex} = 550 nm) of **Zn$_2$-CP2$_m$** (blue broken line) and **Fb$_2$-CP2$_m$** (red line) in $CHCl_3$.

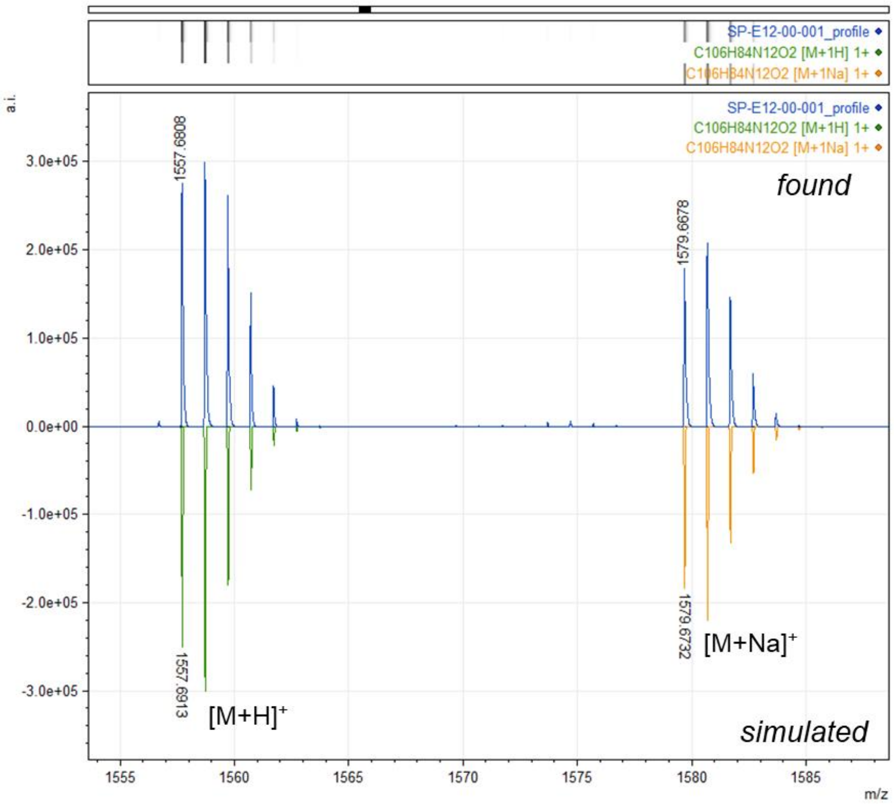

Figure S2. MALDI-TOF mass (matrix: dithranol) of **Fb$_2$-CP2$_m$**. Top: found, bottom: simulated by $[C_{106}H_{85}N_{12}O_2]^+$ ($[M+H]^+$) and $[C_{106}H_{84}N_{12}O_2Na]^+$ ($[M+Na]^+$).

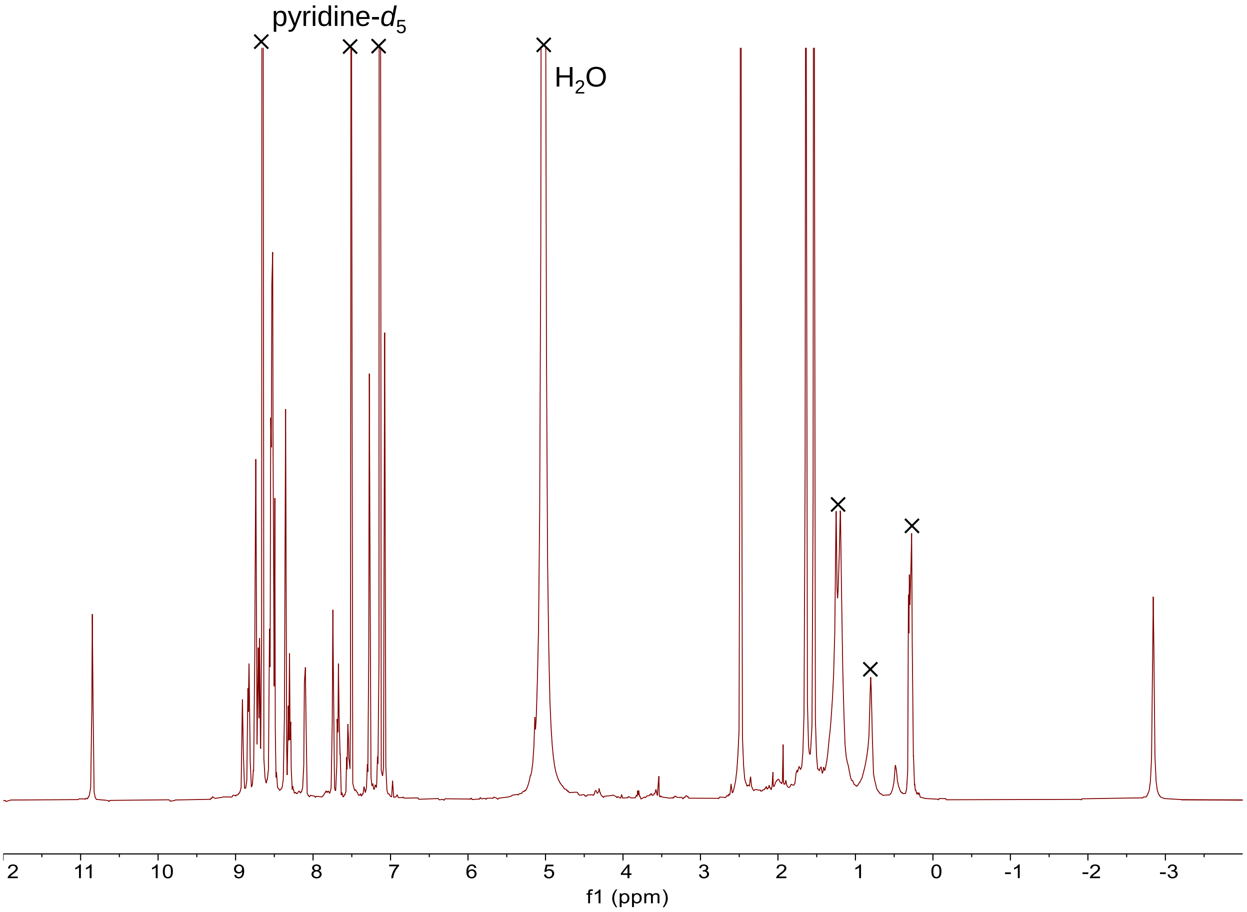

Figure S3. ^{1}H NMR spectrum of **Fb_2-CP2$_m$** in pyridine-d_5 (500 MHz). Cross marks indicate impurities.

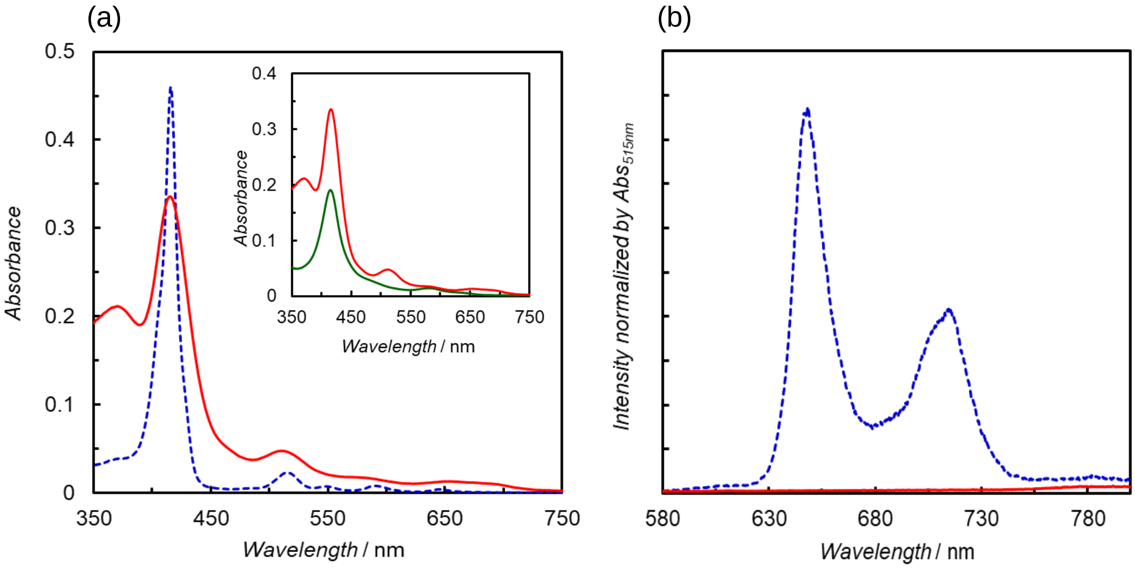

Figure S4. (a) UV–vis absorption spectra and (b) fluorescence spectra (λ_{ex} = 515 nm) of **Fb$_2$-CP2$_m$** (blue broken line) and HCl-treated **Fe$_2$-CP2$_m$** (red line) in $CHCl_3$. The inset shows absorption spectra before (red line) and after (green line) treatment of **Fe$_2$-CP2$_m$** with HCl aqueous solution.

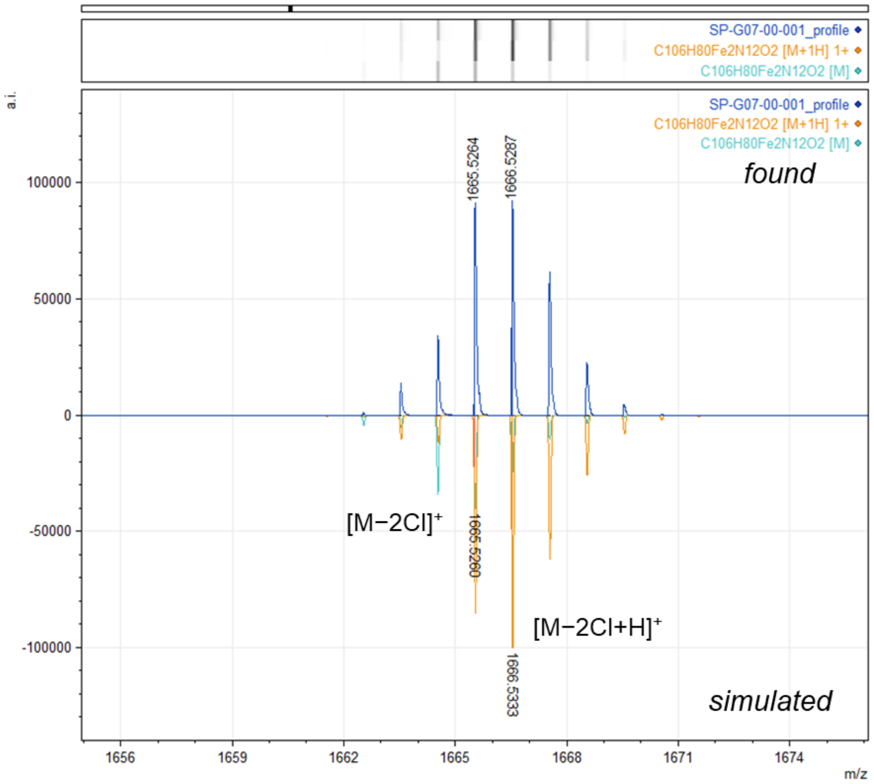

Figure S5. MALDI-TOF mass (matrix: dithranol) of **Fe$_2$-CP2$_m$**. Top: found, bottom: simulated by $[C_{106}H_{80}N_{12}O_2Fe_2]^+$ ($[M-2Cl]^+$) and $[C_{106}H_{81}N_{12}O_2Fe_2]^+$ ($[M-2Cl+H]^+$) .

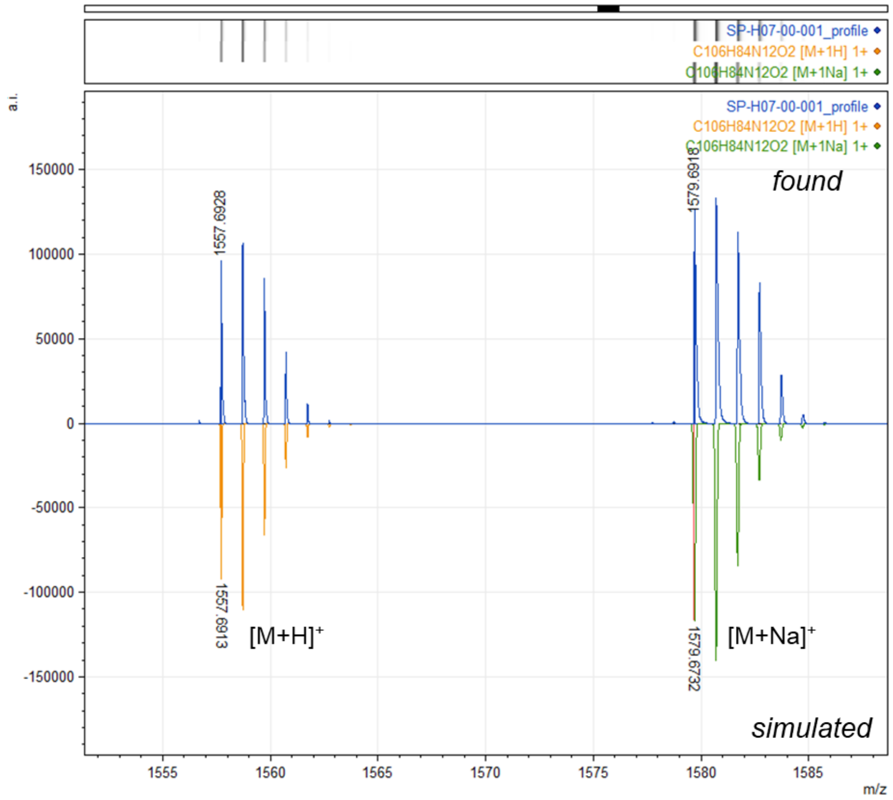

Figure S6. MALDI-TOF mass (matrix: dithranol) of **Fb2-CP2*p***. Top: found, bottom: simulated by $[C_{106}H_{85}N_{12}O_2]^+$ ($[M+H]^+$) and $[C_{106}H_{84}N_{12}O_2Na]^+$ ($[M+Na]^+$).

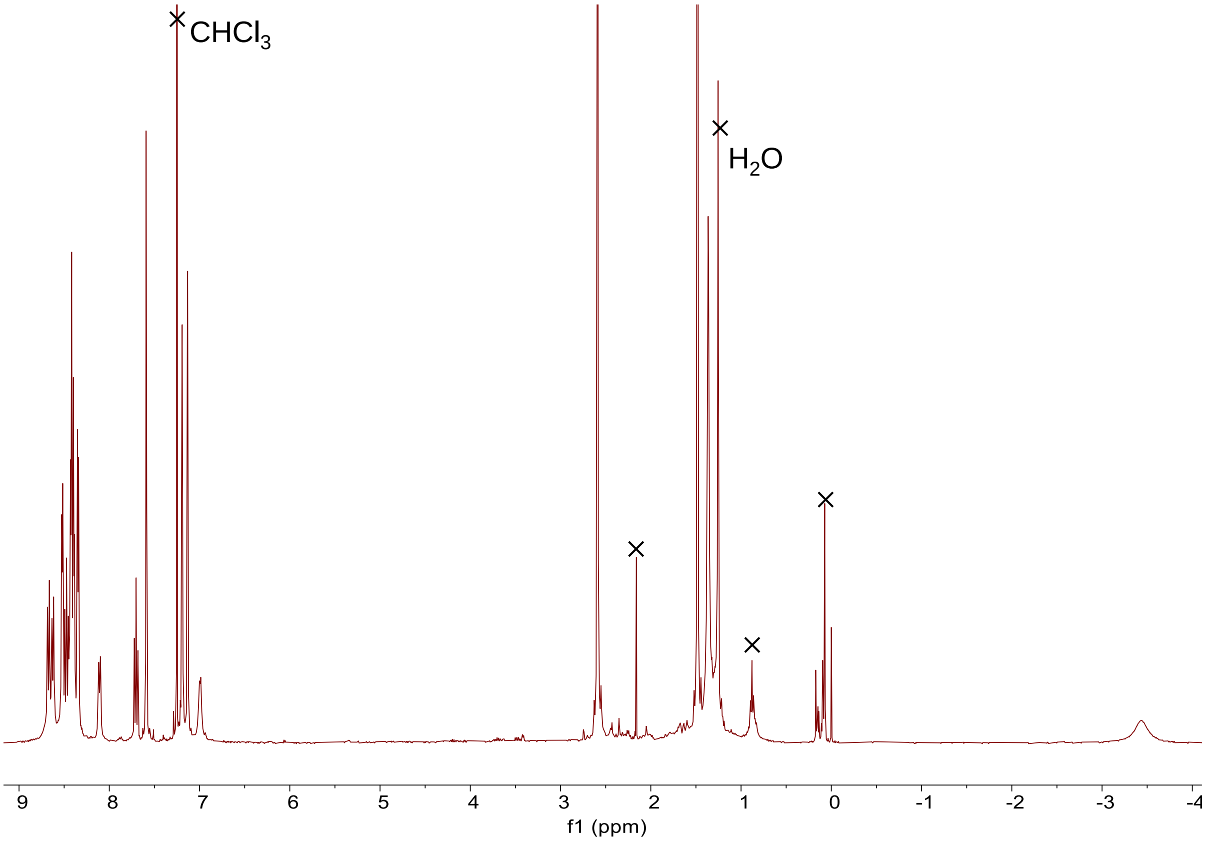

Figure S7. ^{1}H NMR spectrum of **Fb2-CP2*p*** in $CDCl_3$ (400 MHz). Cross marks indicate impurities.

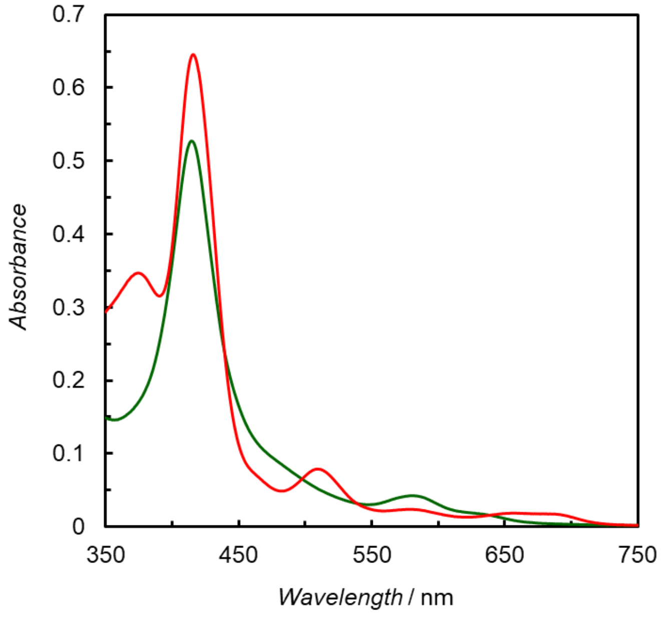

Figure S8. UV–vis absorption spectra of **Fe_2-CP2*p*** in $CHCl_3$ before (red line) and after (green line) treatment with HCl aqueous solution.

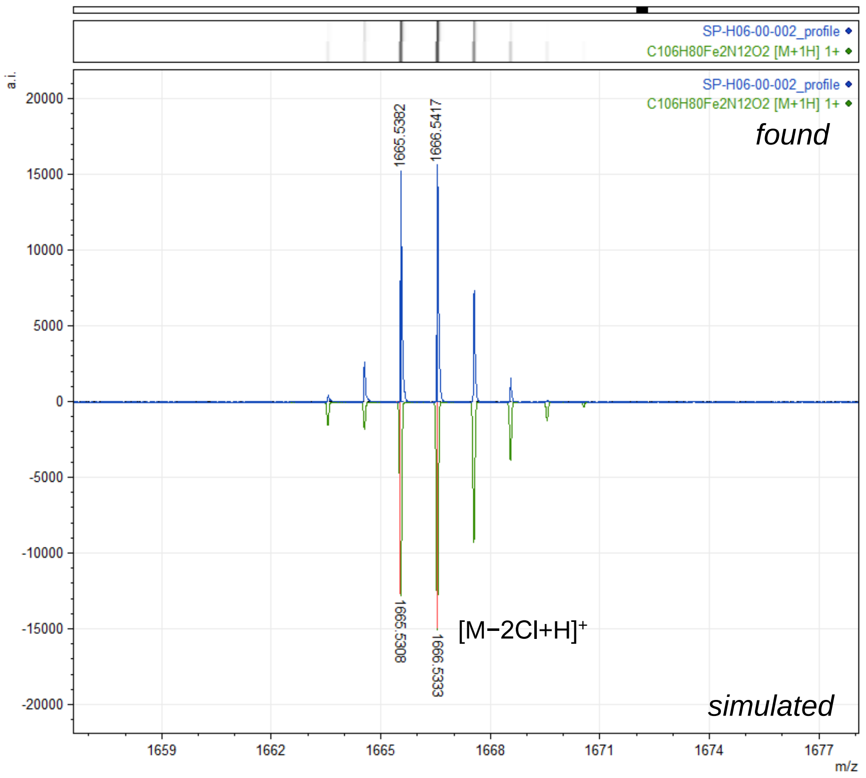

Figure S9. MALDI-TOF mass (matrix: dithranol) of **Fe_2-CP2*p***. Top: found, bottom: simulated by[$C_{106}H_{81}N_{12}O_2Fe_2$]$^+$ ([M−2Cl+H]$^+$) .

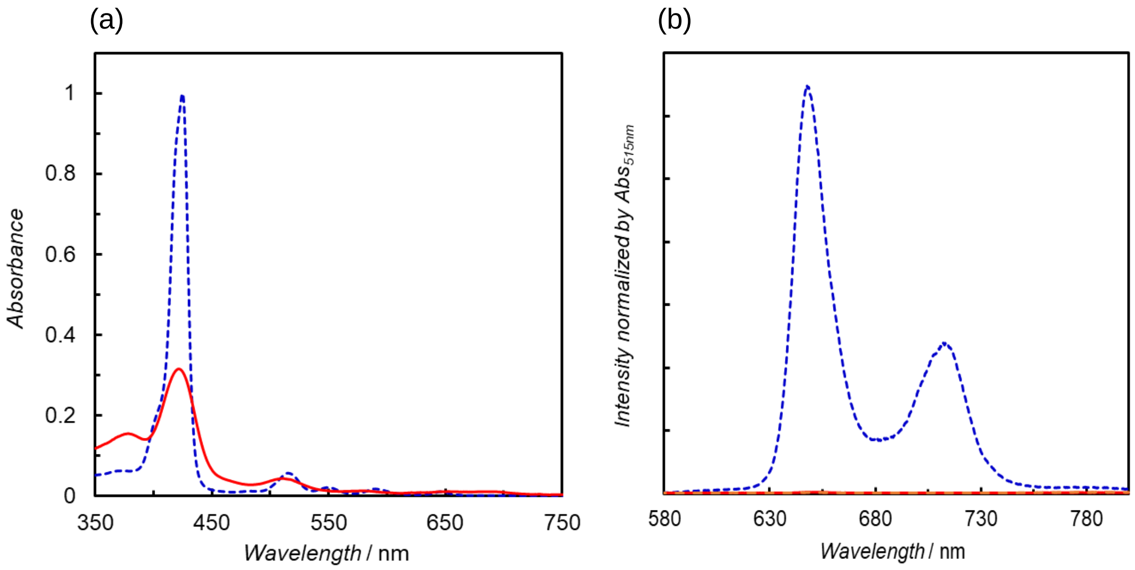

Figure S10. (a) UV–vis absorption spectra and (b) fluorescence spectra (λ_{ex} = 515 nm) of **Fb2-P2** (blue broken line) and brine-treated **Fe2-P2** (red line) in $CHCl_3$.

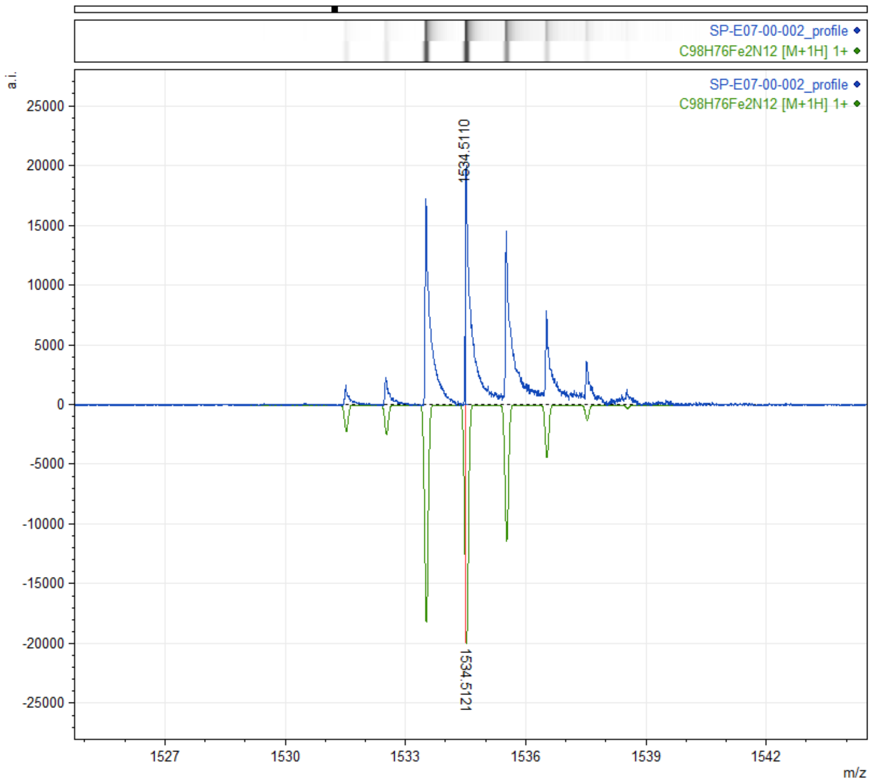

Figure S11. MALDI-TOF mass (matrix: dithranol) of **Fe2-P2**. Top: found, bottom: simulated by $[C_{98}H_{76}Fe_2N_{12}]^+$ ($[M{-}2Boc{-}2Cl{+}H]^+$).

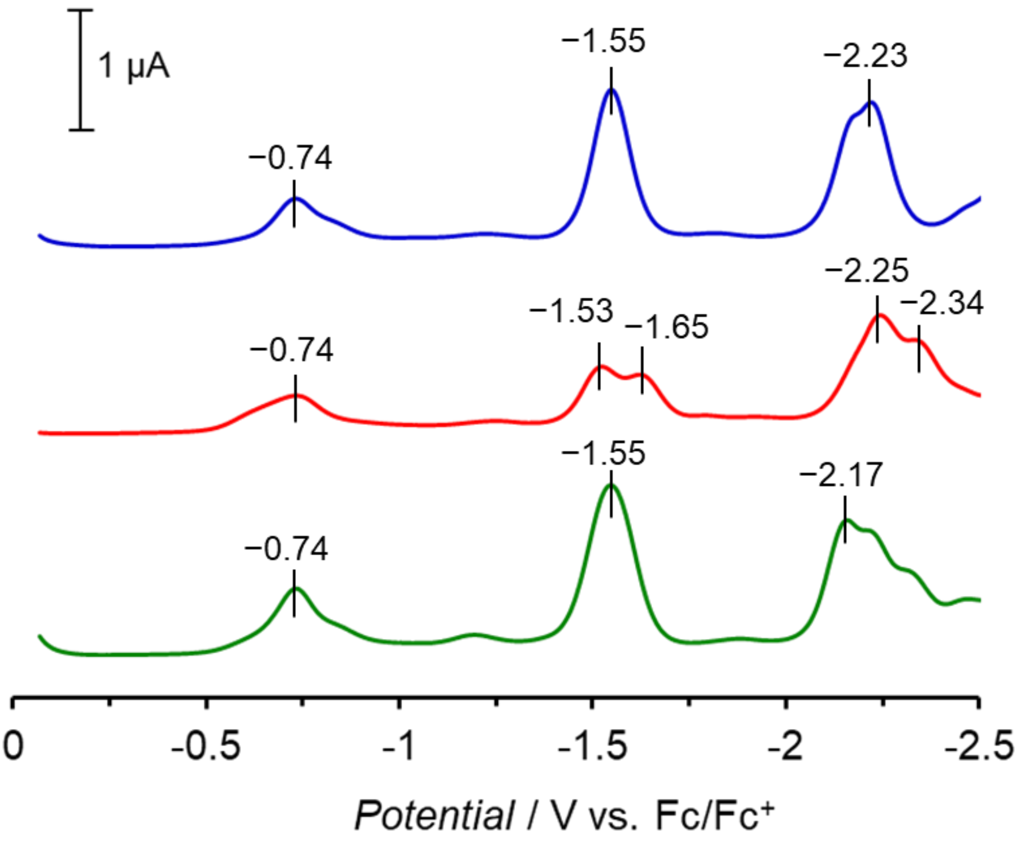

Figure S12. DPVs of (a) **Fe_2-P2**, (b) **Fe_2-CP2*m***, and (c) **Fe_2-CP2*p*** (0.3 mM) collected in dry DMF under an Ar atmosphere with 0.1 M $^{n}Bu_4NPF_6$ as the supporting electrolyte.

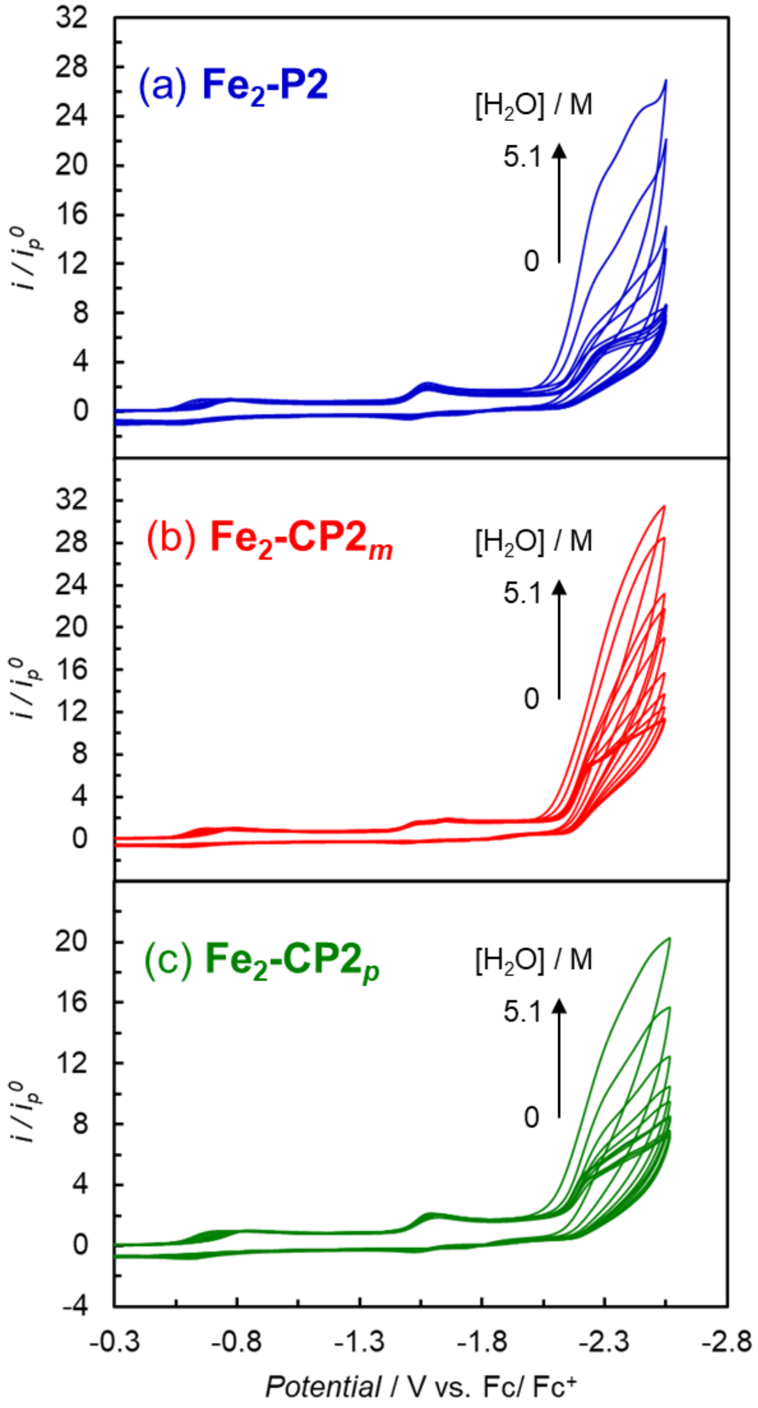

Figure S13. CVs (Scan rate = 100 mV s^{-1}) of (a) **Fe_2-P2**, (b) **Fe_2-CP2*m***, and (c) **Fe_2-CP2*p*** (0.3 mM) collected in CO_2-saturated DMF and 0.1 M $^{n}Bu_4NPF_6$ in the presence of water.

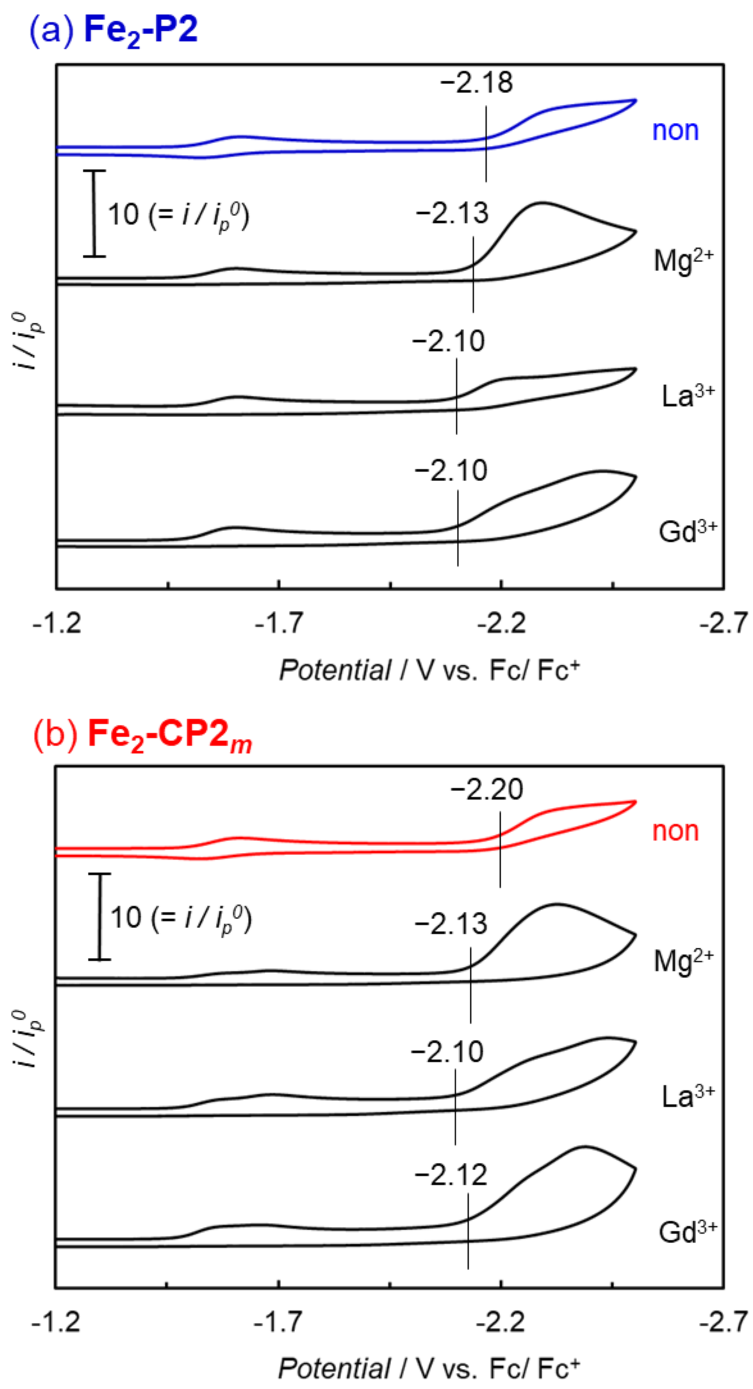

Figure S14. CVs (Scan rate = 100 mV s^{-1}) of (a) **Fe_2-P2** and (b) **Fe_2-CP2*$_m$*** (0.3 mM) collected in CO_2-saturated DMF and 0.1 M $^{n}Bu_4NPF_6$ in the presence of $M(OTf)_n$ (3.0 mM; M = Mg (*n* =2), La and Gd (*n* = 3)).

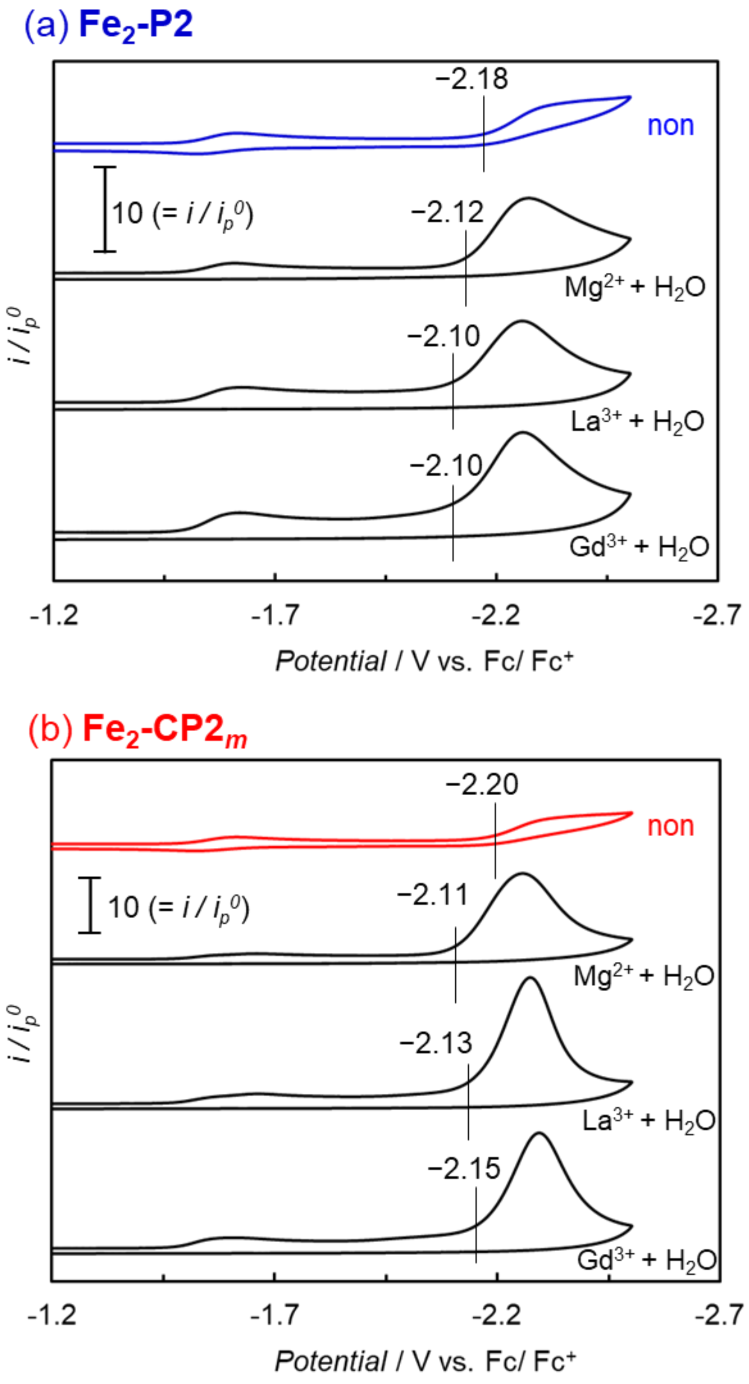

Figure S15. CVs (Scan rate = 100 mV s^{-1}) of (a) **Fe_2-P2** and (b) **Fe_2-CP2*m*** (0.3 mM) in CO_2-saturated DMF and 0.1 M $^n Bu_4NPF_6$ in the presence of $M(OTf)_n$ (3.0 mM; M = Mg (*n* =2), La and Gd (*n* = 3)) and water (1.8 M).

Table S1. Effect of each additive on the ratio of maximum current (i_{max}) to i_p^0

Fe porphyrin [a]	Additive [b]	i_{max} / i_p^0
**Fe_2-P2**	none	6.0
	H_2O (5.1 M)	27
	Mg^{2+}	9.2
	La^{3+}	5.2
	Gd^{3+}	8.5
	Mg^{2} + H_2O (1.8 M)	9.2
	La^{3+} + H_2O (1.8 M)	10
	Gd^{3+} + H_2O (1.8 M)	12
**Fe_2-CP2$_m$**	none	6.0
	H_2O (5.1 M)	32
	Mg^{2+}	9.1
	La^{3+}	8.8
	Gd^{3+}	11
	Mg^{2} + H_2O (1.8 M)	16
	La^{3+} + H_2O (1.8 M)	23
	Gd^{3+} + H_2O (1.8 M)	21
**Fe_2-CP2$_p$**	none	8.5
	H_2O (5.1 M)	20

[a] [Fe porphyrin] = 0.3 mM in CO_2-saturated DMA containing $^nBu_4NPF_6$ (0.1 M). [b] [$M(OTf)_n$] = 0.3 mM.

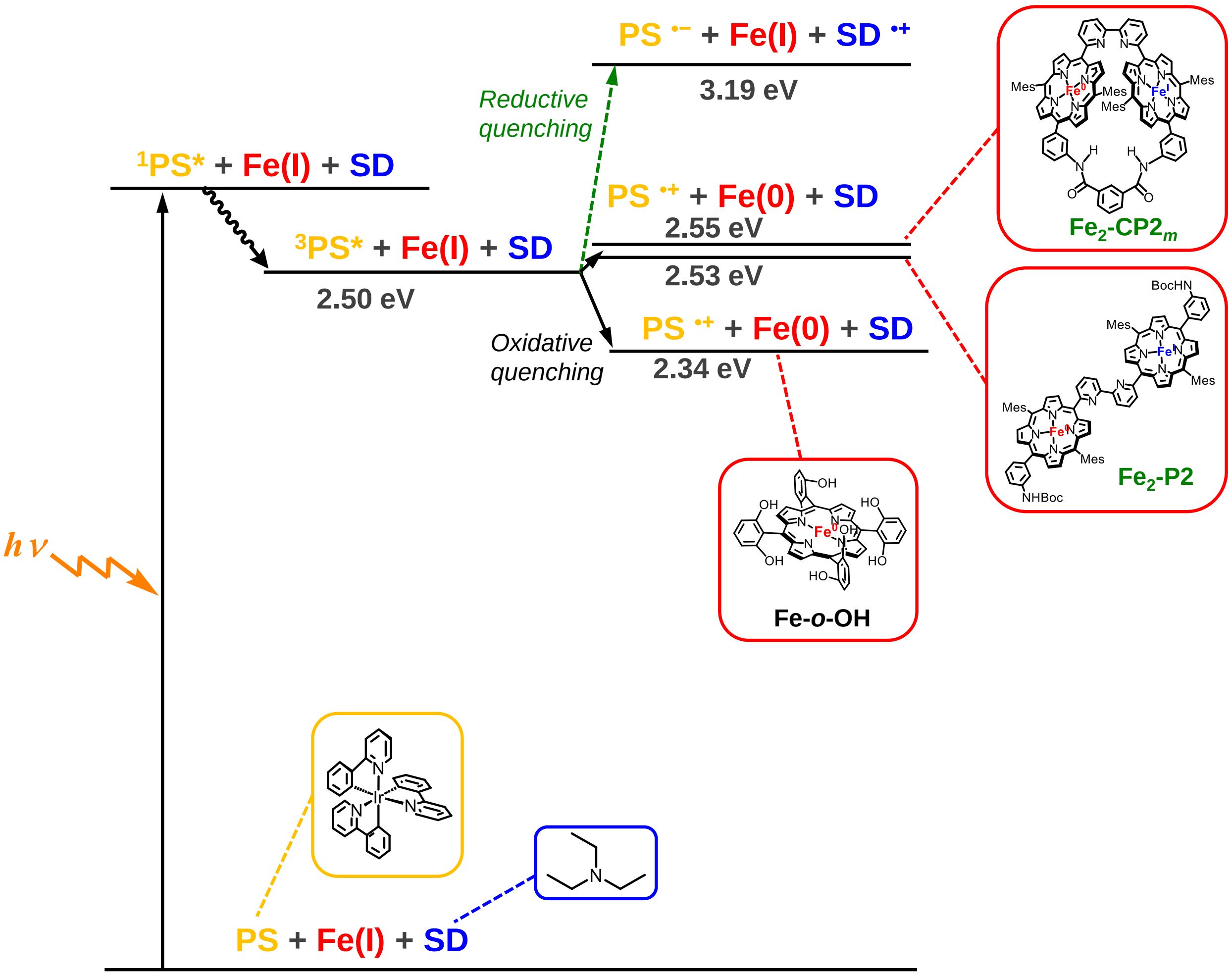

Figure S16. Energy diagram of the photoinduced electron transfer during the formation of Fe(0) species via the oxidative quenching process by either **Fe-*o*-OH** or the Fe porphyrin dimer, when Ir(ppy)$_3$ is used as a photosensitizer (PS) and TEA as a sacrificial donor (SD). The redox potentials, except for **Fe$_2$-P2** and **Fe$_2$-CP2$_m$**, were obtained from the following literature: Anxolabéhère-Mallart, E.; Bonin, J.; Fave, C.; Robert, M., *Dalton Trans.* **2019**, *48*, 5869. The redox potentials corresponding to Fe(I)/Fe(0) for **Fe$_2$-P2** and **Fe$_2$-CP2$_m$** were determined using a value of −2.23 and −2.25 V vs Fc/Fc$^+$, respectively (−1.76 and −1.78 V vs SCE). When using TEA, the reductive quenching is a highly endothermic process.

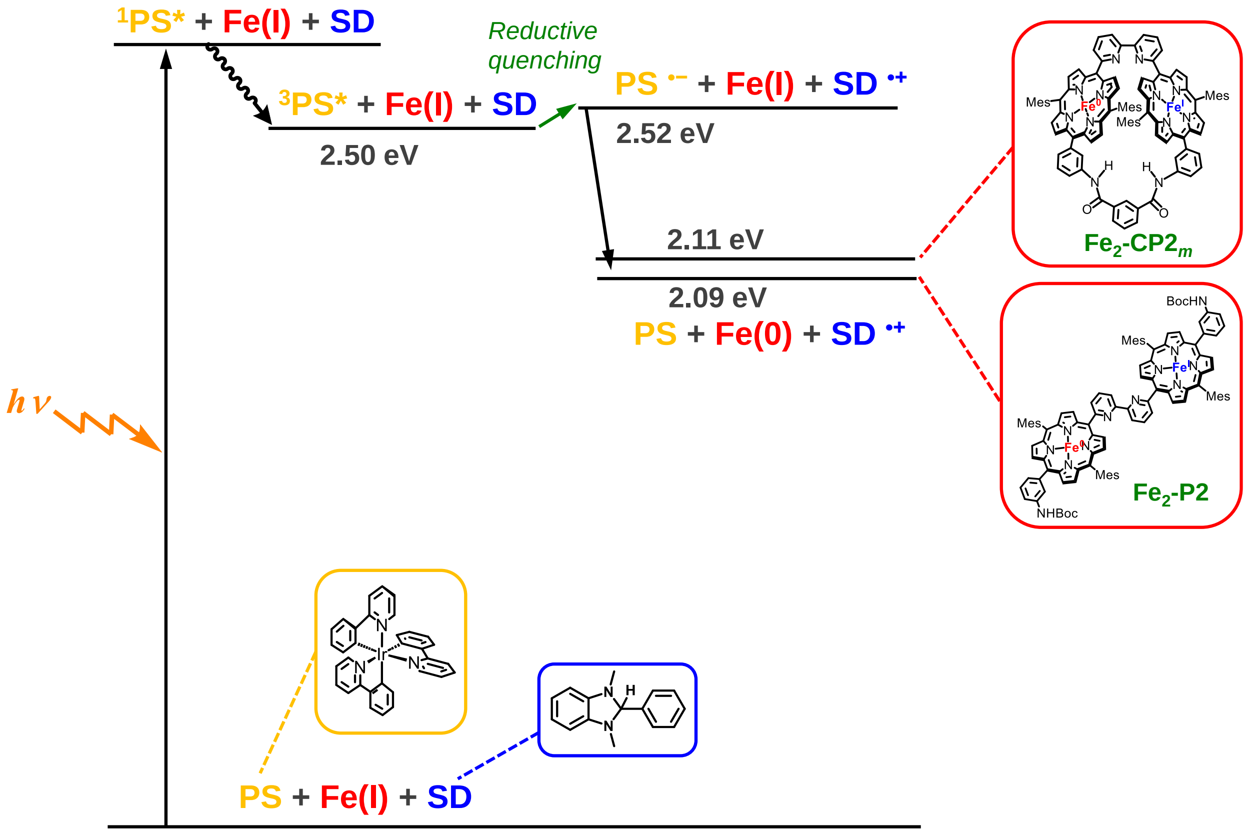

Figure S17. Energy diagram of the photoinduced electron transfer during the formation of Fe(0) species via the reductive quenching process by BIH, when $Ir(ppy)_3$ is used as a photosensitizer (PS) and BIH as a sacrificial donor (SD). The redox potentials, except for **Fe_2-P2** and **Fe_2-CP2*m***, were obtained from the following literature: Anxolabéhère-Mallart, E.; Bonin, J.; Fave, C.; Robert, M., *Dalton Trans.* **2019**, *48*, 5869. The redox potentials corresponding to Fe(I)/Fe(0) for **Fe_2-P2** and **Fe_2-CP2*m*** were determined using a value of −2.23 and −2.25 V vs Fc/Fc^+, respectively (−1.76 and −1.78 V vs SCE).

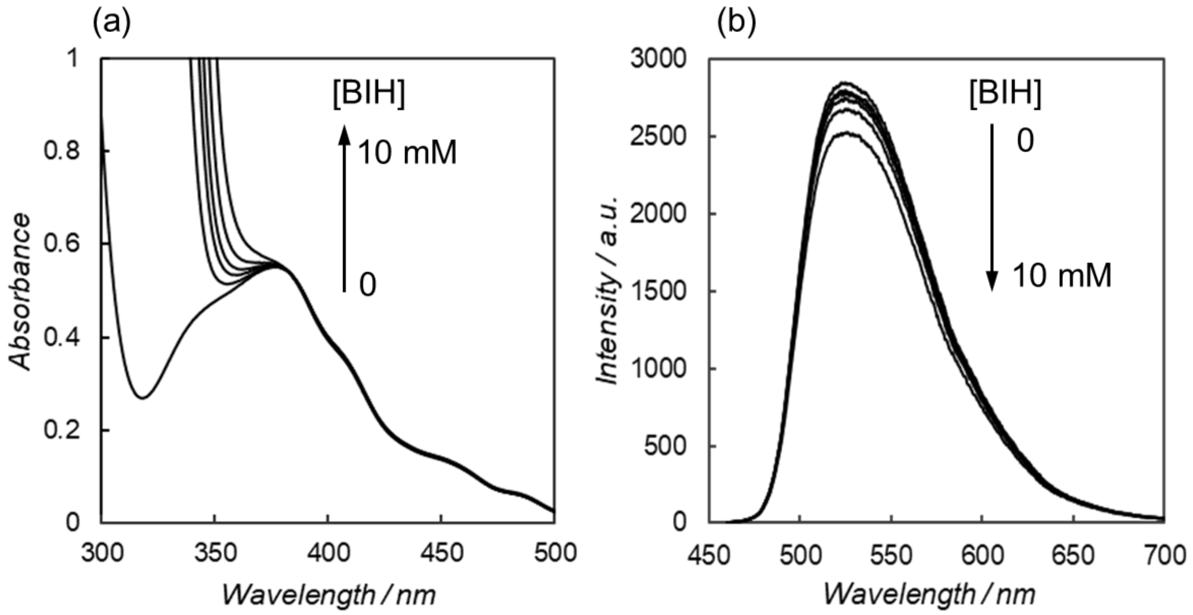

Figure S18. (a) UV–vis absorption spectra and (b) emission spectra (λ_{ex} = 450 nm) of Ir(ppy)$_3$ in Ar-saturated DMA at 298 K in the presence of various amounts of BIH.

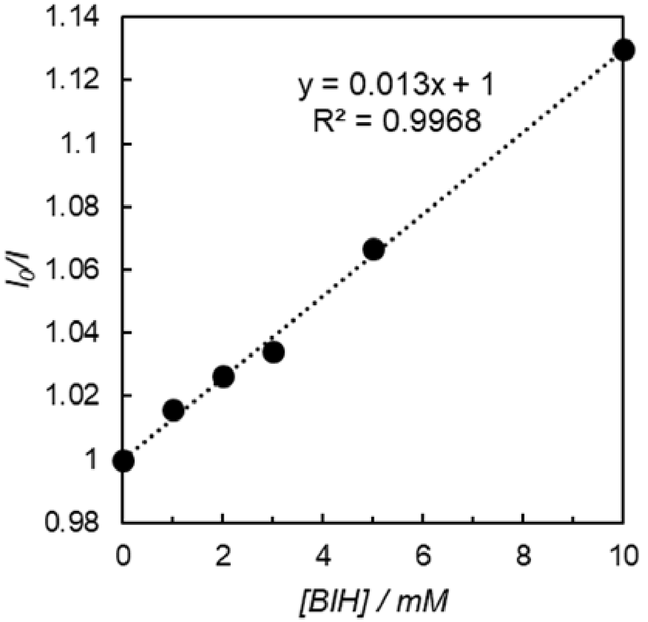

Figure S19. Stern–Volmer plot of emission quenching of Ir(ppy)$_3$ by BIH. From the slope of the plot (K_{SV} = 13 M^{-1}) and the emission lifetime (τ_{em}), the quenching rate constant (k_q) is obtained ($k_q = K_{SV}/\tau_{em}$). Assuming a phosphorescence lifetime of Ir(ppy)$_3$ as 1.9 μs (Dedeian, K.; Djurovich, P. I.; Garces, F. O.; Carlson, G.; Watts, R. J., *Inorg. Chem.* **1991**, *30*, 1685), the estimated value of k_q is 6.8×10^6 M^{-1} s^{-1}. This value is considerably smaller than the rate constant of the diffusion-controlled process (~10^9 M^{-1} s^{-1}) and is consistent with the fact that the electron transfer process is an endothermic process, as illustrated in Figure S17.

Table S2. Photocatalytic reaction using Fe-*o*-OH in acetonitrile (2 mL)[a]

Entry	Cat (2 μM)	Gas	PS (0.2 mM)	SD (10 mM)	Time (min)	TON[b]	
						H_2	CO
1	**Fe-*o*-OH**	CO_2	Ir(ppy)$_3$	BIH	30	2	80
2	**Fe-*o*-OH**	CO_2	Ir(ppy)$_3$	BIH	60	2	84
3	**Fe-*o*-OH**	CO_2	Ir(ppy)$_3$	BIH	120	3	104
4	**Fe-*o*-OH**	Ar	Ir(ppy)$_3$	BIH	120	8	N.D.[c]
5	–	CO_2	Ir(ppy)$_3$	BIH	120	N.D.[c]	N.D.[c]

[a] Irradiation at 450 nm using a merry-go-round apparatus equipped with LED lamps (Input power: 10 mW). [b] TON was calculated based on the Fe porphyrin. [c] Not detected.

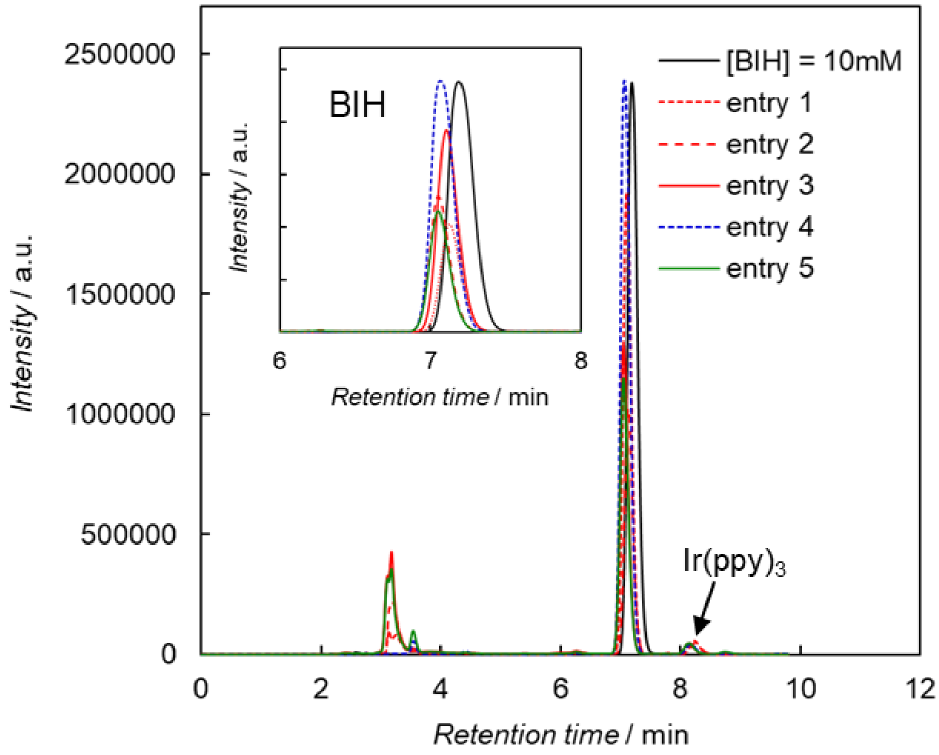

Figure S20. HPLC charts (column: TSKgel ODS-100S, eluent: acetonitrile/H_2O = 4/1 (v/v), flow rate: 1.0 mL/min, detection: 320 nm) of the reaction solutions containing BIH (10 mM) and Ir(ppy)$_3$ (0.2 mM) before and after irradiation. The reaction conditions and the concentrations are provided in Table S2. The initial peak at ca 7 min corresponding to BIH was significantly decreased during irradiation under a CO_2 atmosphere.

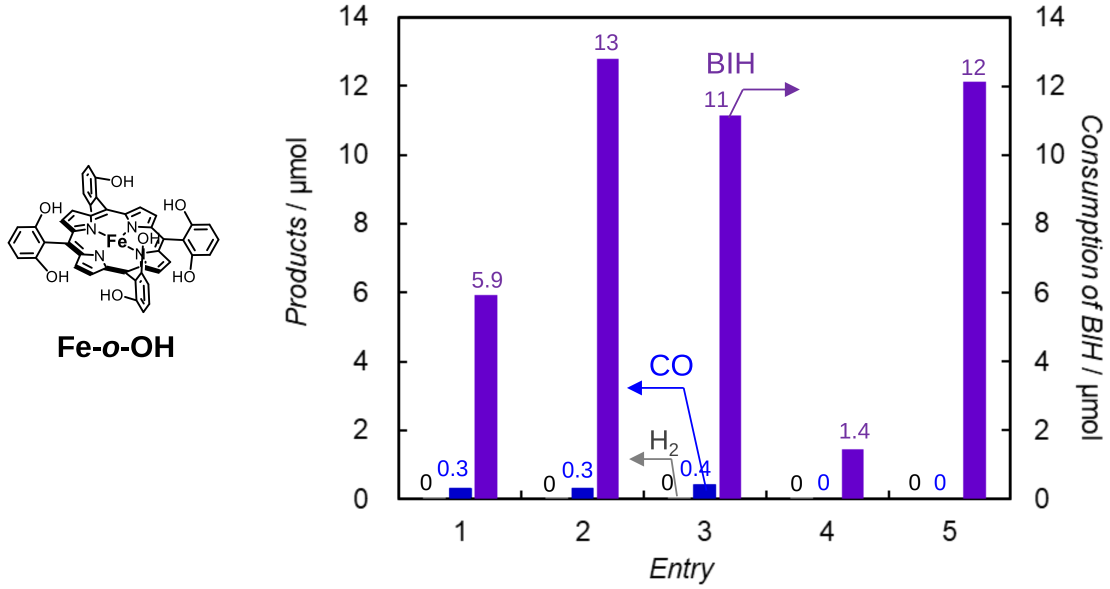

Figure S21. Comparison of the reduction products and BIH consumption in Table S2. The initial amount of BIH before light irradiation was 20 µmol. The consumed amount of BIH was estimated from the decrease in the peak at a retention time of ca. 7 min in Figure S20. The consumption of BIH was significantly higher than the CO production. Moreover, in the condition without the catalyst and CO production, the amount of BIH significantly decreased under a CO_2 atmosphere.

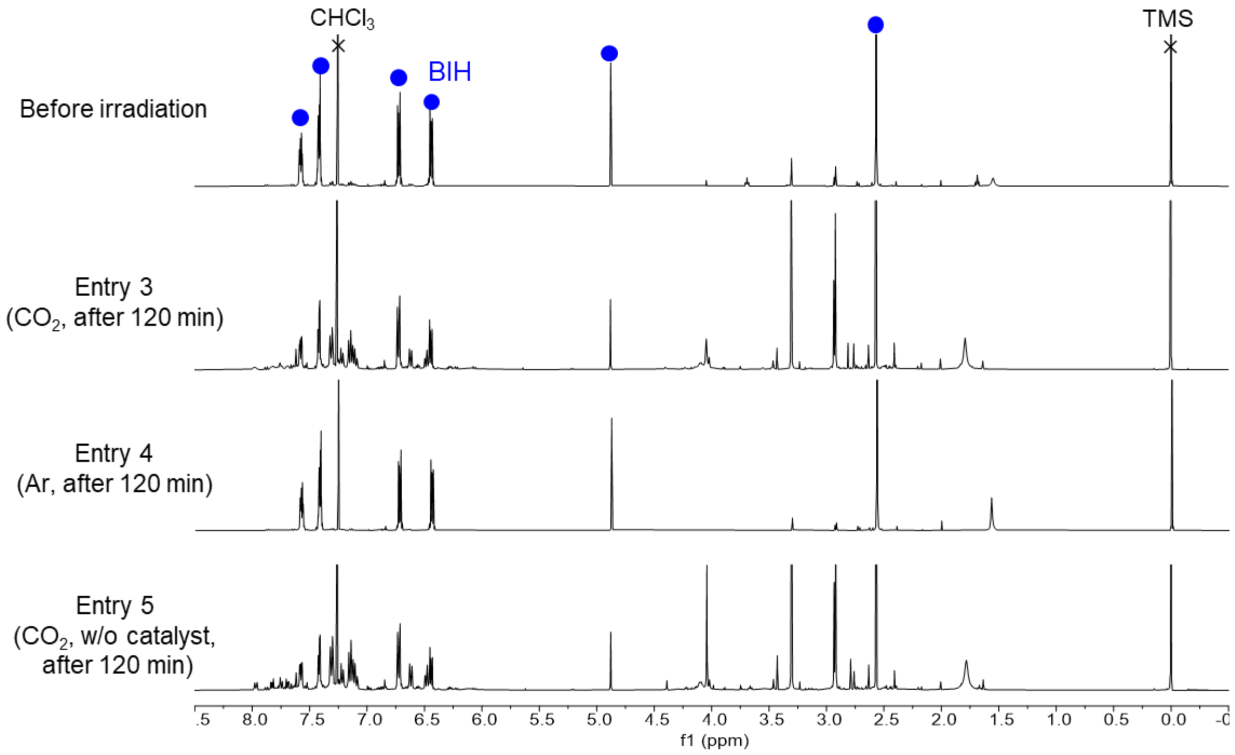

Figure S22. ^1H NMR spectra (400 MHz, $CDCl_3$) of the reaction solutions containing BIH (10 mM) and $Ir(ppy)_3$ (0.2 mM) before and after irradiation. The reaction conditions are provided in Table S2. Unidentified BIH decomposition products were observed in entries 2 and 5. These decomposition products were not observed under an Ar atmosphere (entry 4).

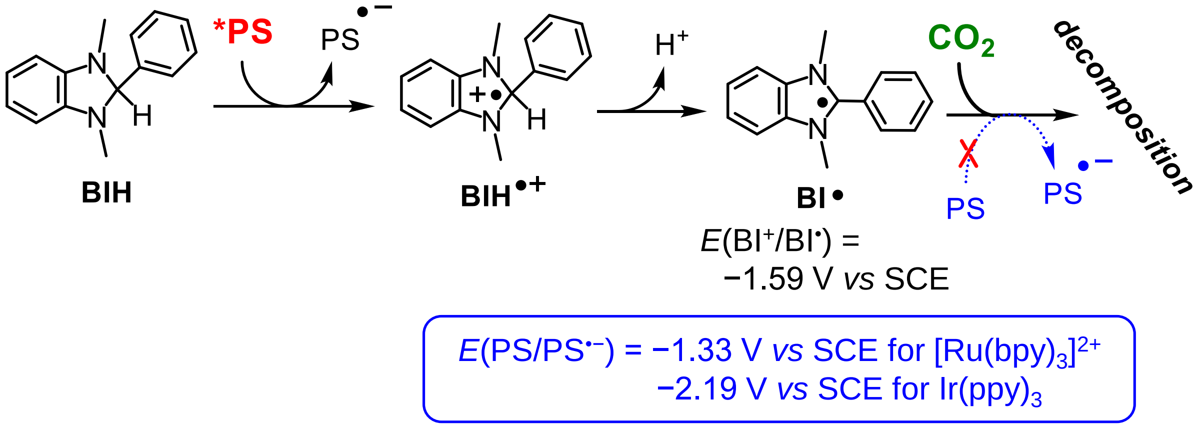

Figure S23. Decomposition of BIH during irradiation in the presence of PS and CO_2. The redox potentials of BI˙, $[Ru(bpy)_3]^{2+}$ and $Ir(ppy)_3$ were obtained from the following literatures: Zhu, X.-Q.; Zhang, M.-T.; Yu, A.; Wang, C.-H.; Cheng, J.-P., *J. Am. Chem. Soc.* **2008**, *130*, 2501; Anxolabéhère-Mallart, E.; Bonin, J.; Fave, C.; Robert, M., *Dalton Trans.* **2019**, *48*, 5869.

Table S3. Effects of solvents and additives on photocatalytic reaction using Fe-*o*-OH[a]

Entry	Solvent and additive	TON[b]	
		H_2	CO
1	acetonitrile	2	84
2	acetonitrile : TEOA = 5 : 1 (v/v)	32	N.D.[c]
3	acetonitrile and 5 vol% TEA	18	16
4	DMA	1	200
5	DMA : TEOA = 5 : 1 (v/v)	6	8
6	DMA and 5 vol% TEA	2	270

[a] Irradiation at 450 nm for 60 min using a merry-go-round apparatus equipped with LED lamps (Input power: 10 mW) to CO_2-saturated solutions (2 mL) containing **Fe-*o*-OH** (2 μM) as a catalyst, $Ir(ppy)_3$ (0.2 mM) as a photosensitizer, and BIH (10 mM) as a sacrificial electron donor. [b] TON was calculated based on the Fe porphyrin. [c] Not detected.

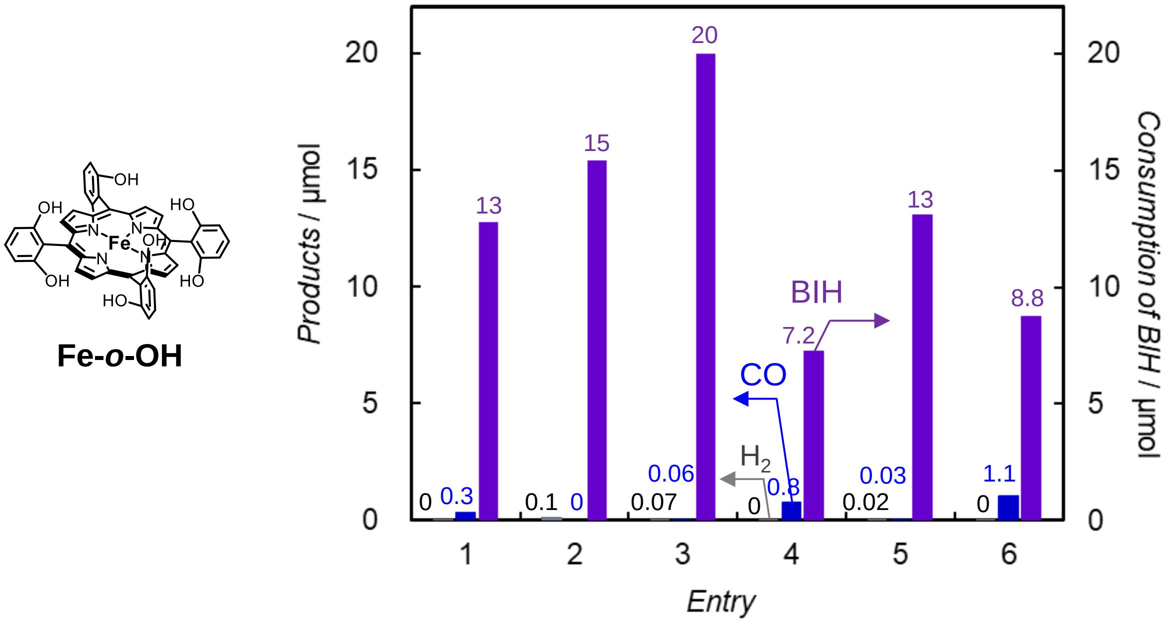

Figure S24. Comparison of the reduction products and BIH consumption in Table S3. The initial amount of BIH before light irradiation was 20 μmol. The consumed amount of BIH was estimated using HPLC. The reaction conditions and the concentrations are provided in Table S3.

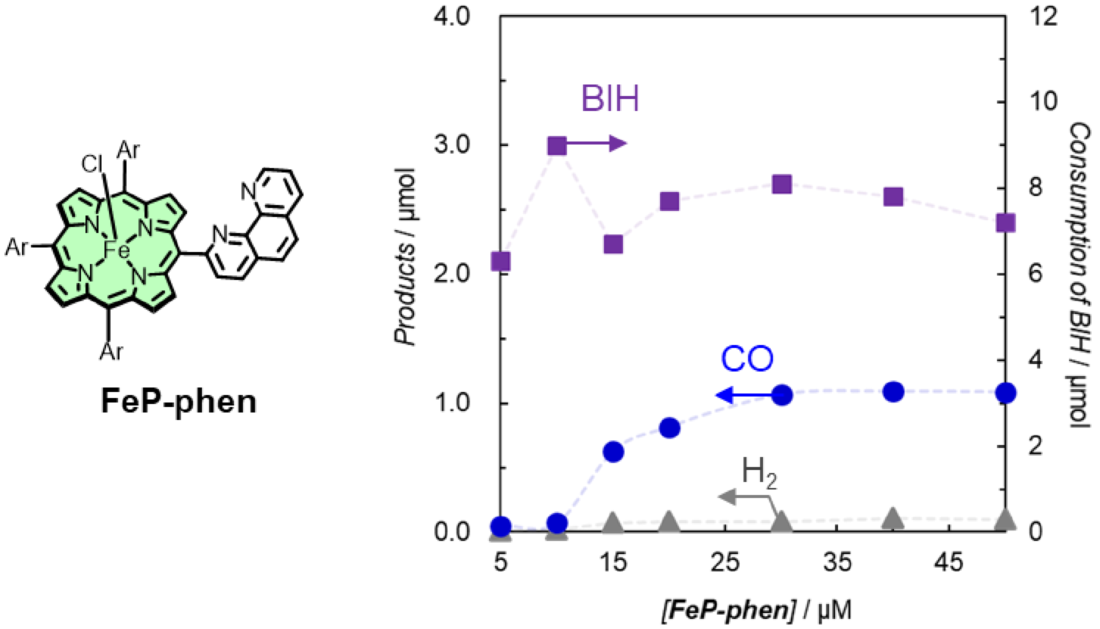

Figure S25. Plots of the amounts of the reduction products and the consumption of BIH after 60 min of irradiation at 450 nm using a merry-go-round apparatus equipped with LED lamps (Input power: 10 mW) versus the concentration of **FeP-phen** in CO_2-saturated DMA (2 mL) in the presence of $Ir(ppy)_3$ (0.2 mM) and BIH (10 mM). No methane was detected. The consumed amount of BIH was estimated using HPLC.

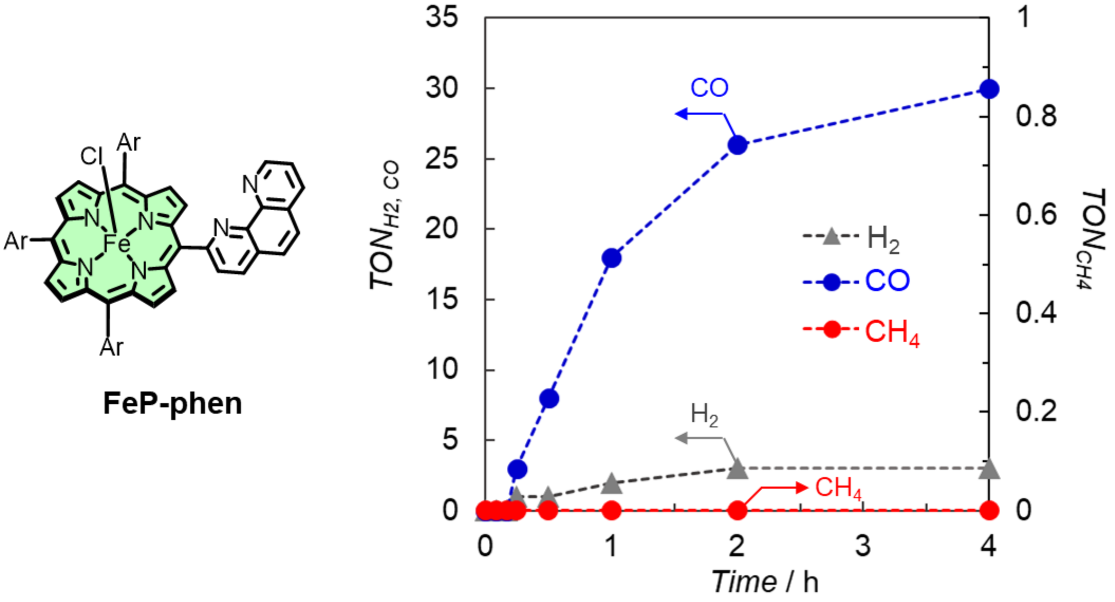

Figure S26. Time dependence of the reduction products during the irradiation of CO_2-saturated DMA solutions (2.0 mL) containing **FeP-phen** (30 µM) in the presence of BIH (10 mM) and Ir(ppy)$_3$ (0.2 mM) at 450 nm using a merry-go-round apparatus equipped with LED lamps (Input power: 10 mW).

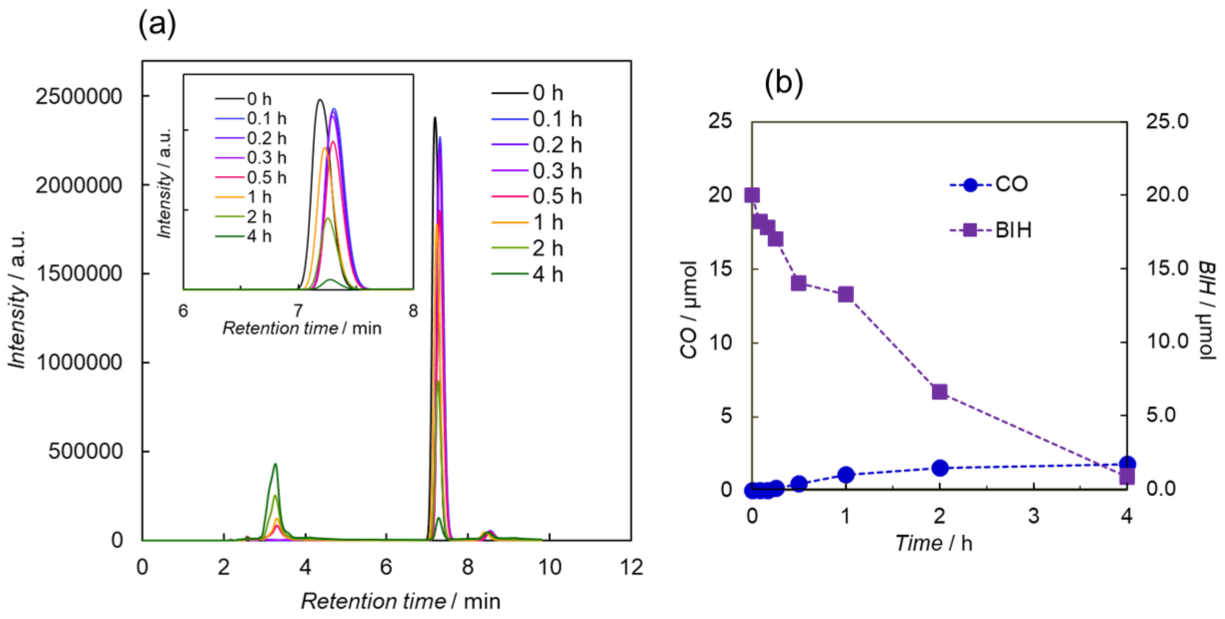

Figure S27. (a) HPLC charts (column: TSKgel ODS-100S, eluent: acetonitrile/H_2O = 4/1 (v/v), flow rate: 1.0 mL/min, detection: 320 nm) of the resulting solutions after irradiation in Figure S26. (b) Time dependence of the remaining amount of BIH determined using HPLC.

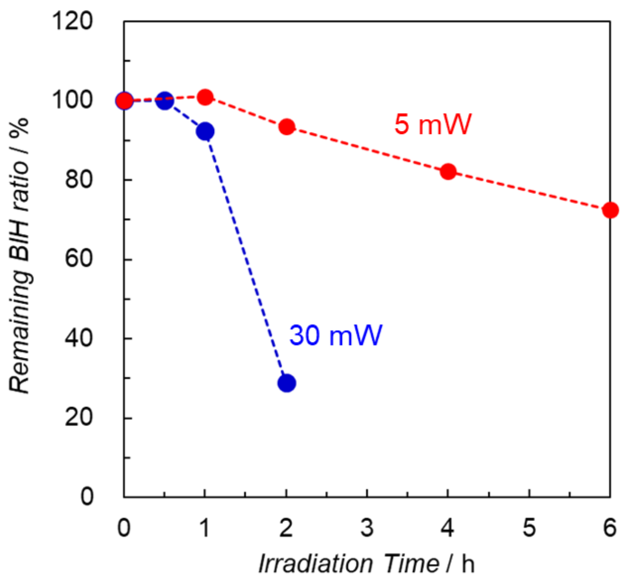

Figure S28. Time dependence of the remaining amount of BIH as per irradiation intensity (Input power: 30 mW or 5 mW) in blank CO_2-saturated DMA solutions containing BIH (10 mM) and Phen2 (1 mM) during irradiation at 420 nm using a merry-go-round apparatus equipped with LED lamps.

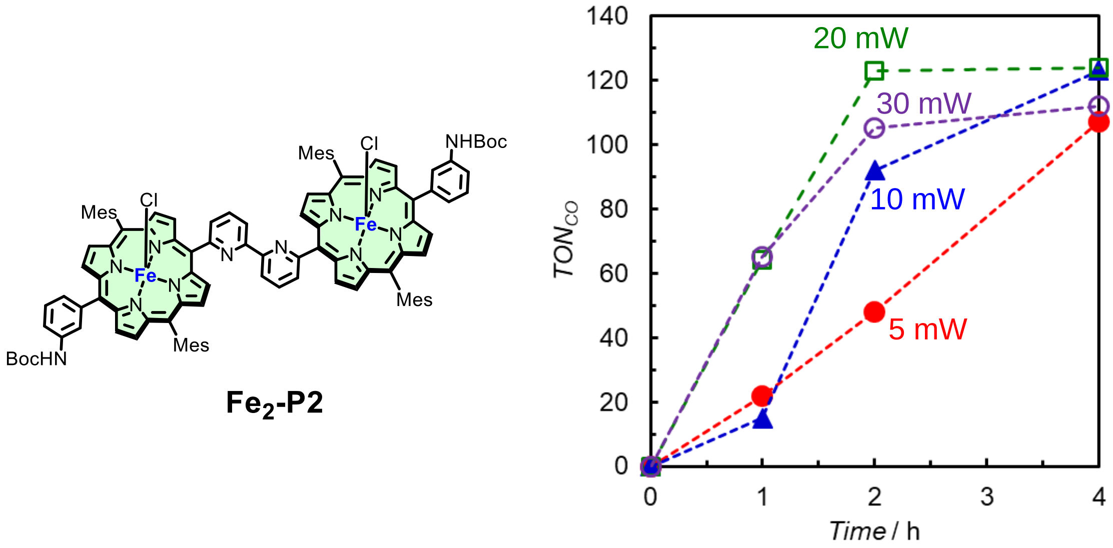

Figure S29. Dependence of CO production on light intensity at 420 nm in CO_2-saturated DMA solutions (2.0 mL) containing **Fe_2-P2** (10 μM), BIH (10 mM), and Phen2 (1 mM), using a merry-go-round apparatus equipped with LED lamps (Input power = light intensity).

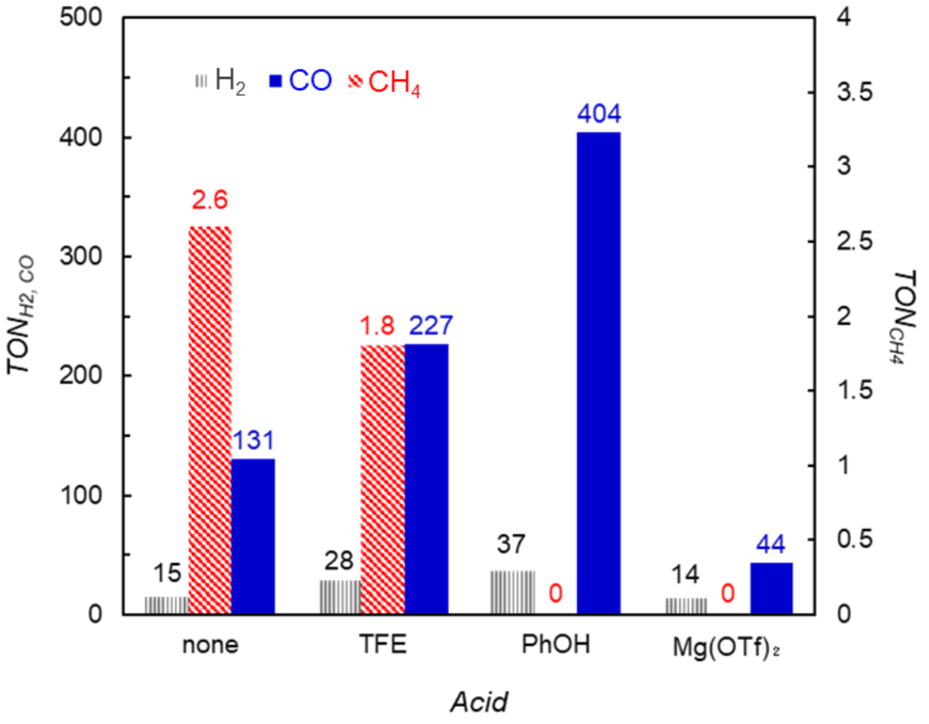

Figure S30. TONs of CO, H_2, and CH_4 during irradiation at 420 nm for 18 h in CO_2-saturated DMA solutions (2.0 mL) containing **Fe2-CP2*p*** (10 μM), BIH (10 mM), and Phen2 (1 mM) in the presence of acids ([TFE] = [PhOH] = 0.1 M; [Mg(OTf)2] = 1 mM), using a merry-go-round apparatus equipped with LED lamps (Input power: 5 mW).

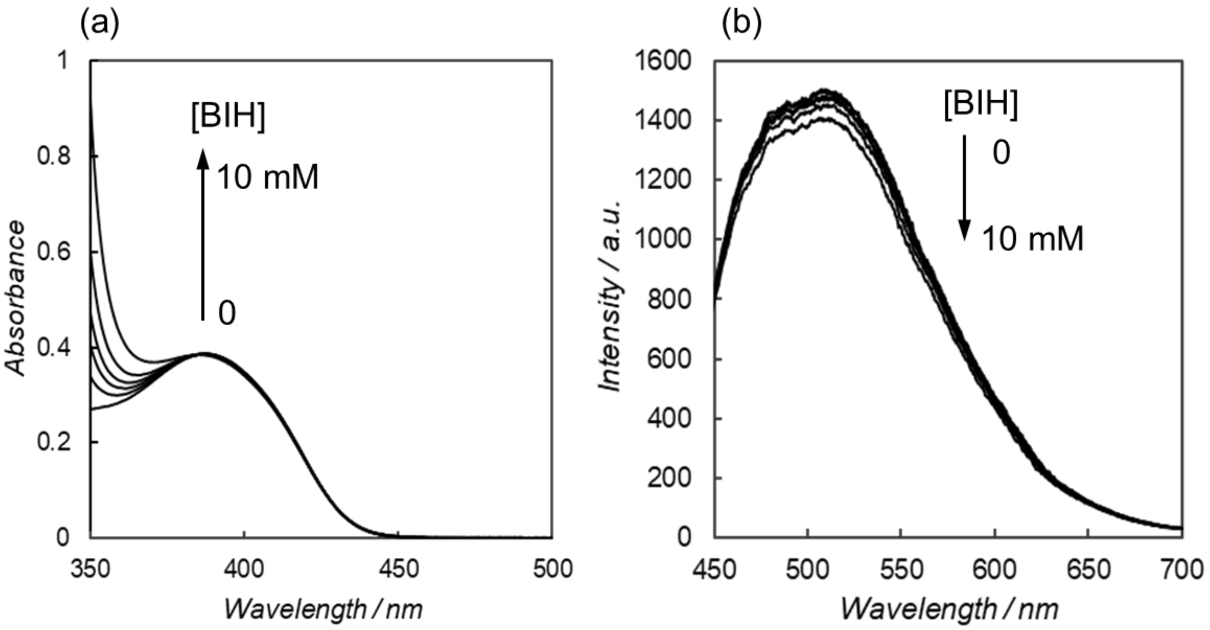

Figure S31. (a) UV–vis absorption spectra and (b) fluorescence spectra (λ_{ex} = 420 nm) of Phen2 in Ar-saturated DMA at 298 K in the presence of various amounts of BIH.

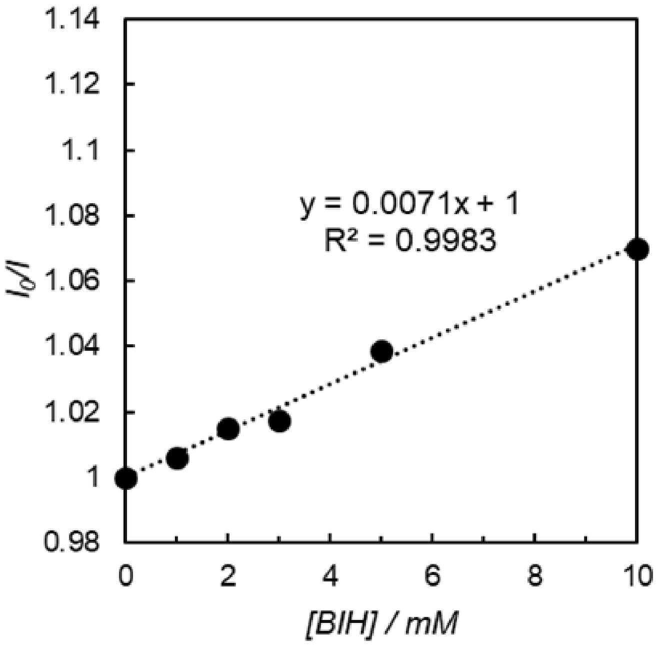

Figure S32. Stern–Volmer plot of emission quenching of Phen2 by BIH. From the slope of the plot (K_{SV} = 7 M^{-1}) and the emission lifetime (τ_{em}), the quenching rate constant (k_q) is obtained ($k_q = K_{SV}/\tau_{em}$). Assuming a fluorescence lifetime of Phen2 as 5.2 ns (McCarthy, B. G.; Pearson, R. M.; Lim, C.-H.; Sartor, S. M.; Damrauer, N. H.; Miyake, G. M., *J. Am. Chem. Soc.* **2018**, *140*, 5088), the estimated value of k_q is 1.3×10^9 M^{-1} s^{-1}. This value is similar to the rate constant of the diffusion-controlled process (~10^9 M^{-1} s^{-1}), indicating that the electron transfer process from BIH to the excited singlet state of Phen2 is a highly exothermic process.

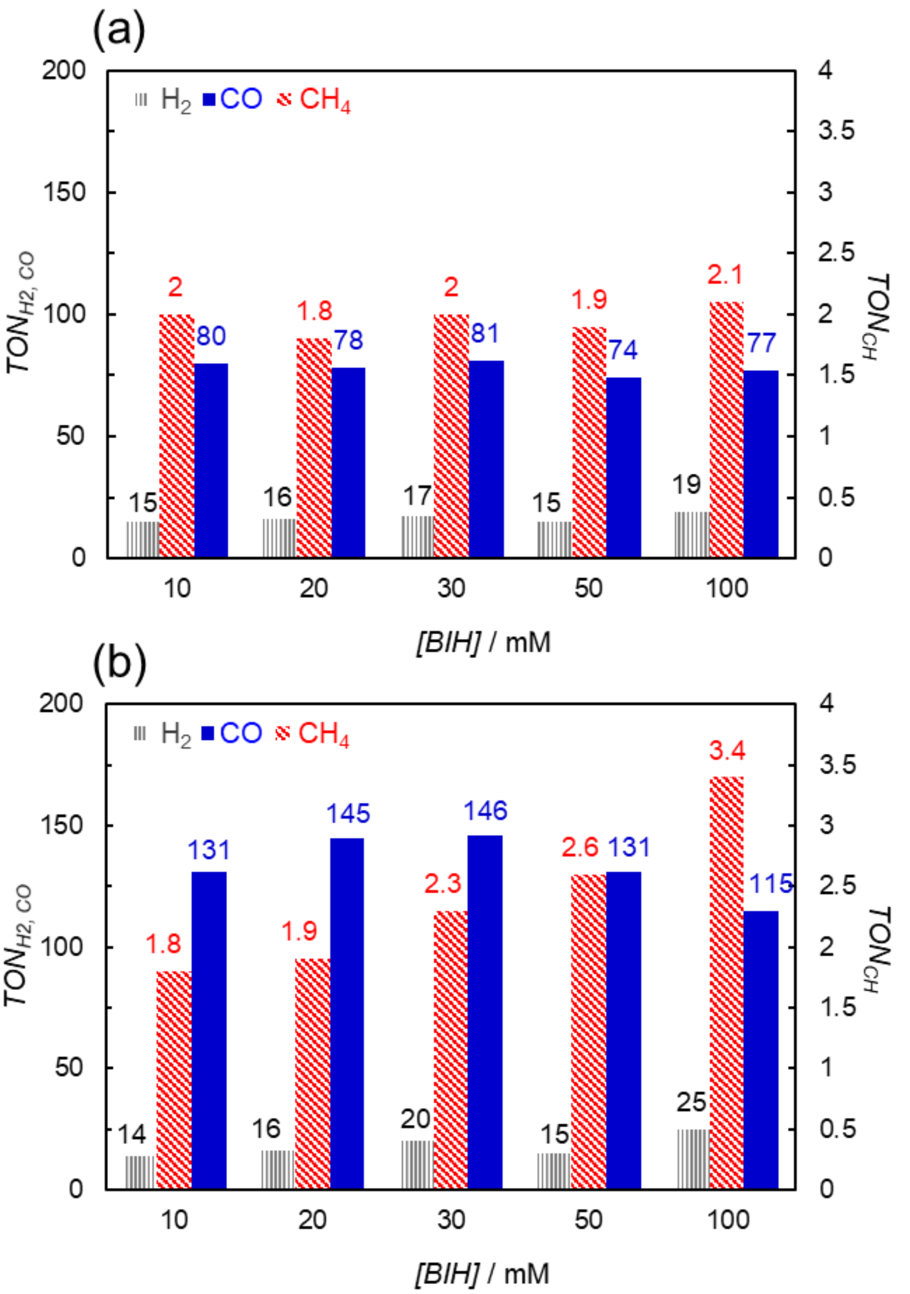

Figure S33. Relationship between the reduction products and the initial concentration of BIH during irradiation at 420 nm for (a) 4 h and (b) 18 h in CO_2-saturated DMA solutions (2.0 mL) containing **Fe2-CP2*p*** (10 μM) and Phen2 (1 mM), using a merry-go-round apparatus equipped with LED lamps (Input power: 5 mW).

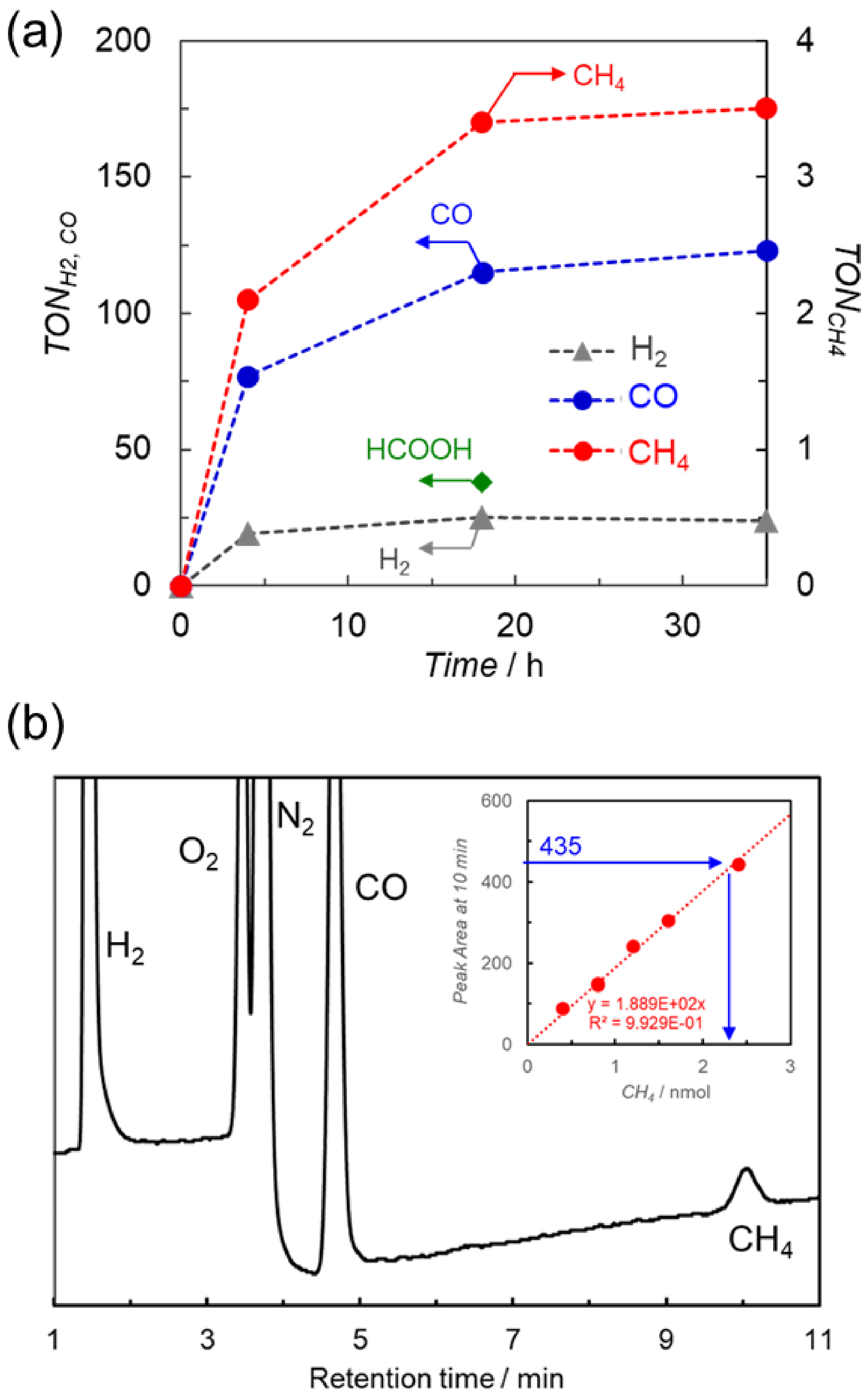

Figure S34. (a) Time dependence of the reduction products during the irradiation of CO_2-saturated DMA solutions (2.0 mL) containing **Fe_2-CP2*$_p$*** (10 μM) in the presence of BIH (100 mM) and Phen2 (1 mM) at 420 nm using a merry-go-round apparatus equipped with LED lamps (Input power: 5 mW). (b) Gas chromatogram of the gaseous reaction products after the irradiation for 35 h. The inset shows the calibration curve obtained using standard CH_4 gas.

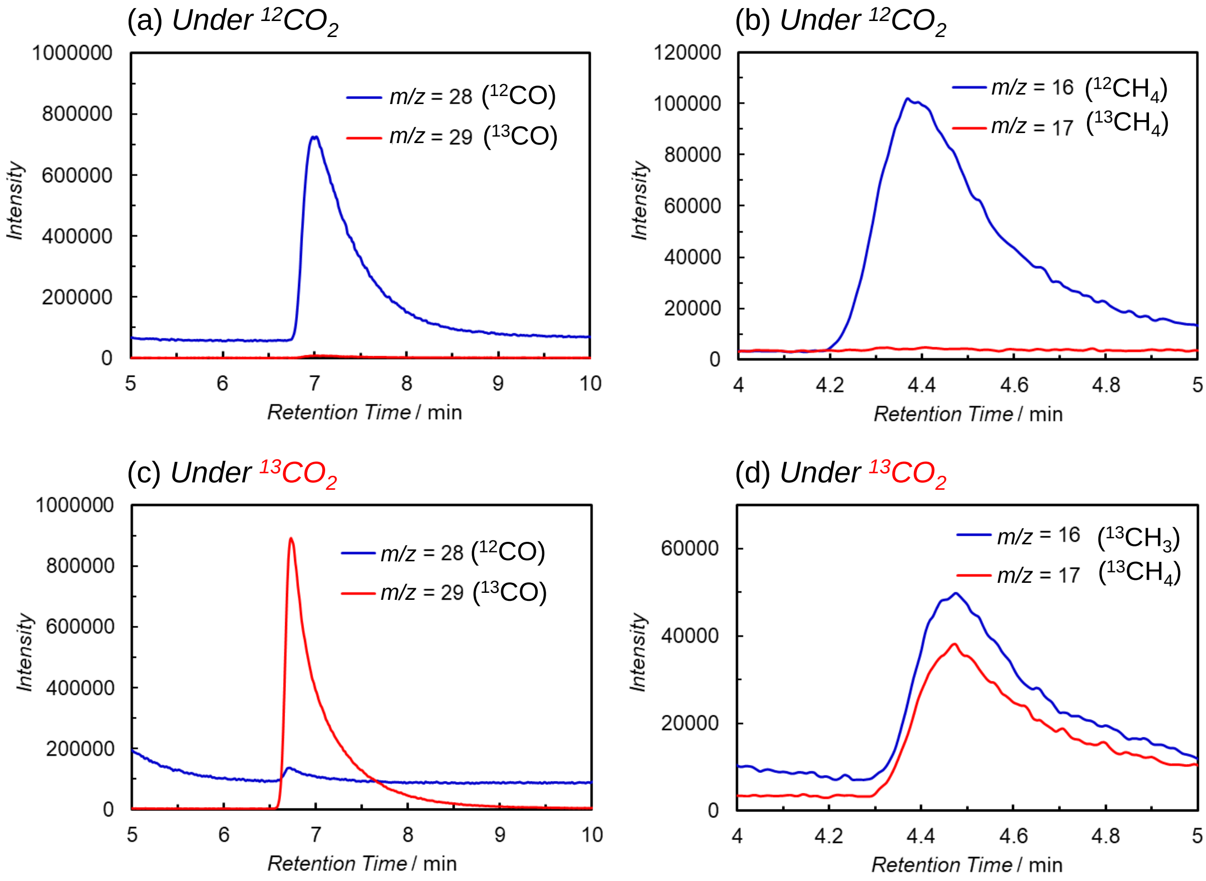

Figure S35. Gas chromatograms of the resulting gas-phase products after irradiation at 420 nm for 14 h under (a), (b) $^{12}CO_2$ or (c), (d) $^{13}CO_2$ atmosphere in DMA solutions (4.0 mL) containing **Fe_2-CP2$_p$** (10 μM), BIH (100 mM), and Phen2 (1 mM) obtained using mass spectroscopy. The irradiation was conducted using a merry-go-round apparatus equipped with LED lamps (Input power: 5mW).

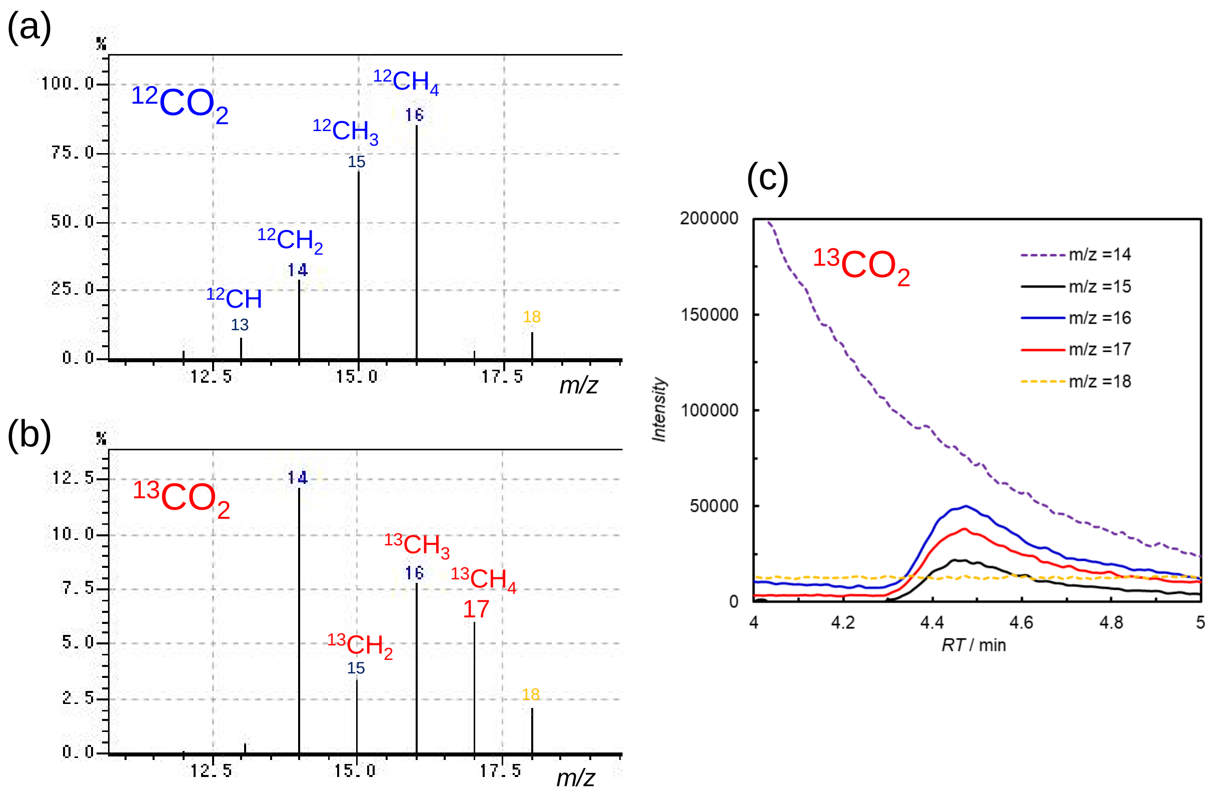

Figure S36. Mass spectra of CH_4 generated under (a) $^{12}CO_2$ and (b) $^{13}CO_2$ atmosphere. (c) Gas chromatogram of the products obtained from the reaction under $^{13}CO_2$ atmosphere, plotted for each *m/z*. The plots at *m/z* =14 and 18 do not show clear peaks and are considered to be derived from substances other than CH_4.

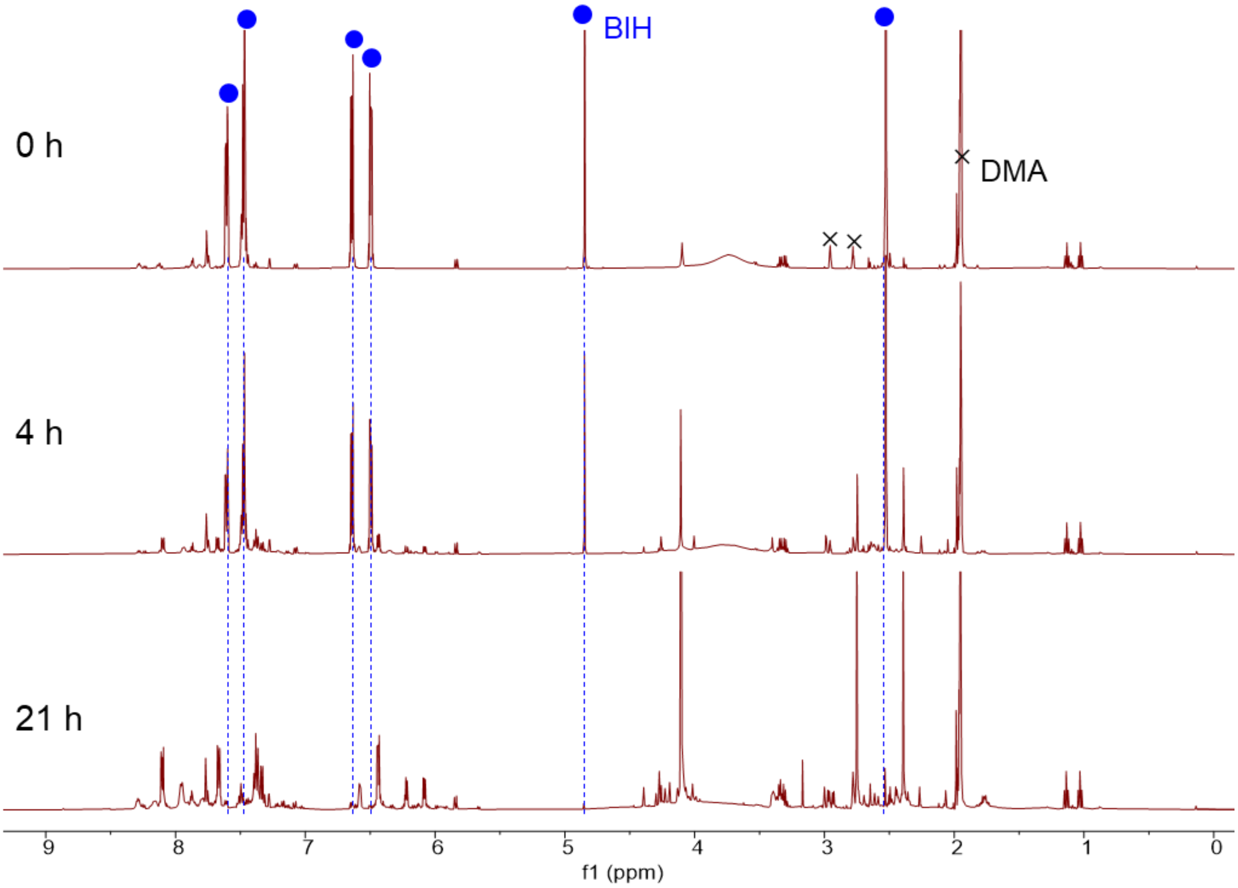

Figure S37. ^{1}H NMR spectra (500 MHz, DMA-*d*$_9$, 0.5 mL) of the reaction solutions containing **Fe$_2$-CP2*$_p$*** (100 μM), BIH (100 mM), and Phen2 (1 mM) during irradiation at 420 nm (5 mW) under a $^{13}CO_2$ atmosphere. Cross marks indicate impurities.

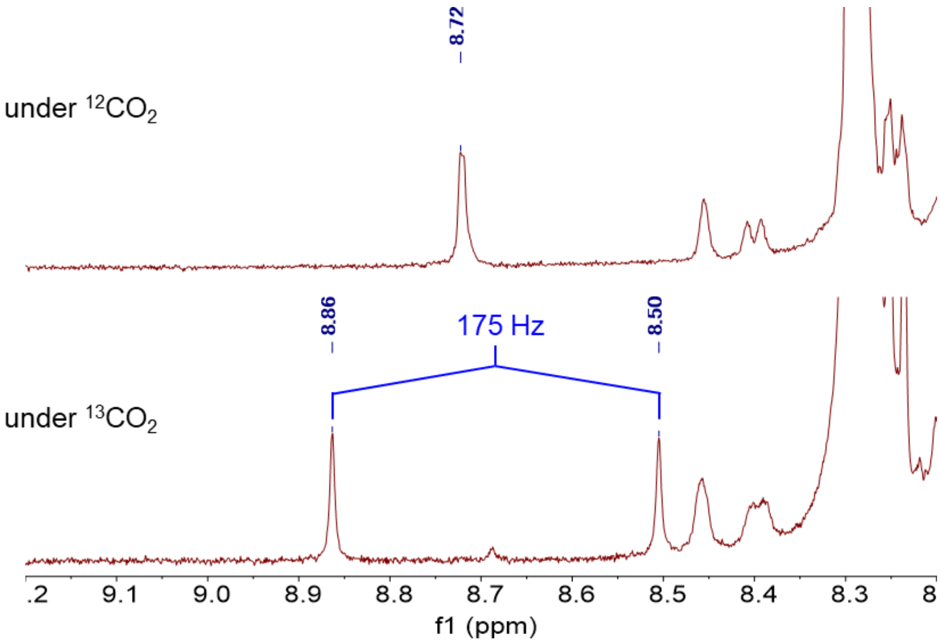

Figure S38. Comparison of the ^{1}H NMR spectra (500 MHz, DMA-*d*$_9$, 0.5 mL) of reaction solutions containing **Fe$_2$-CP2*$_p$*** (100 μM), BIH (100 mM), and Phen2 (1 mM) after irradiation at 420 nm for 21 h under $^{12}CO_2$ and $^{13}CO_2$ atmospheres.

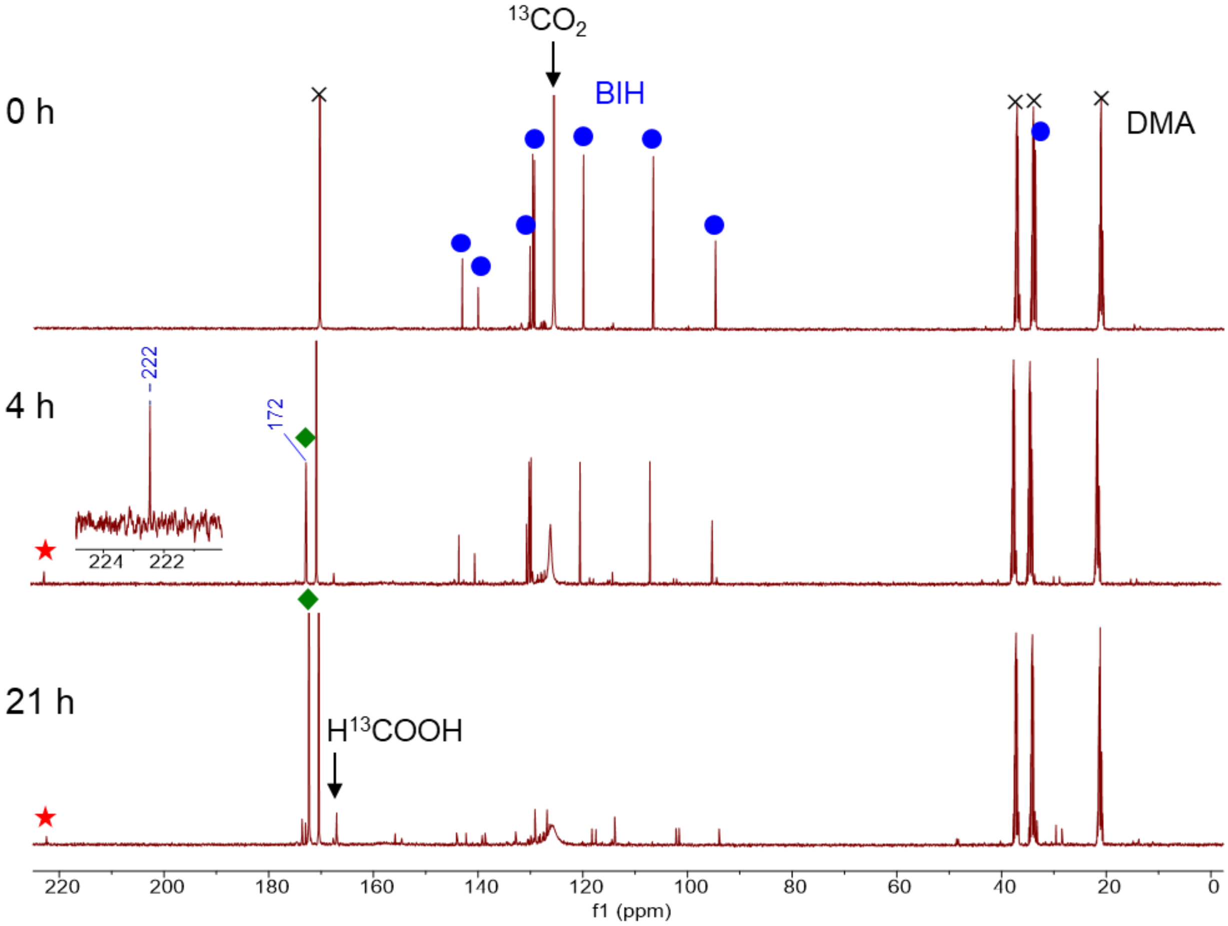

Figure S39. ^{13}C NMR spectra (175 MHz, DMA-d_9, 0.5 mL) of the reaction solutions containing **Fe$_2$-CP2$_p$** (100 μM), BIH (100 mM), and Phen2 (1 mM) during irradiation at 420 nm (5 mW) under a $^{13}CO_2$ atmosphere.

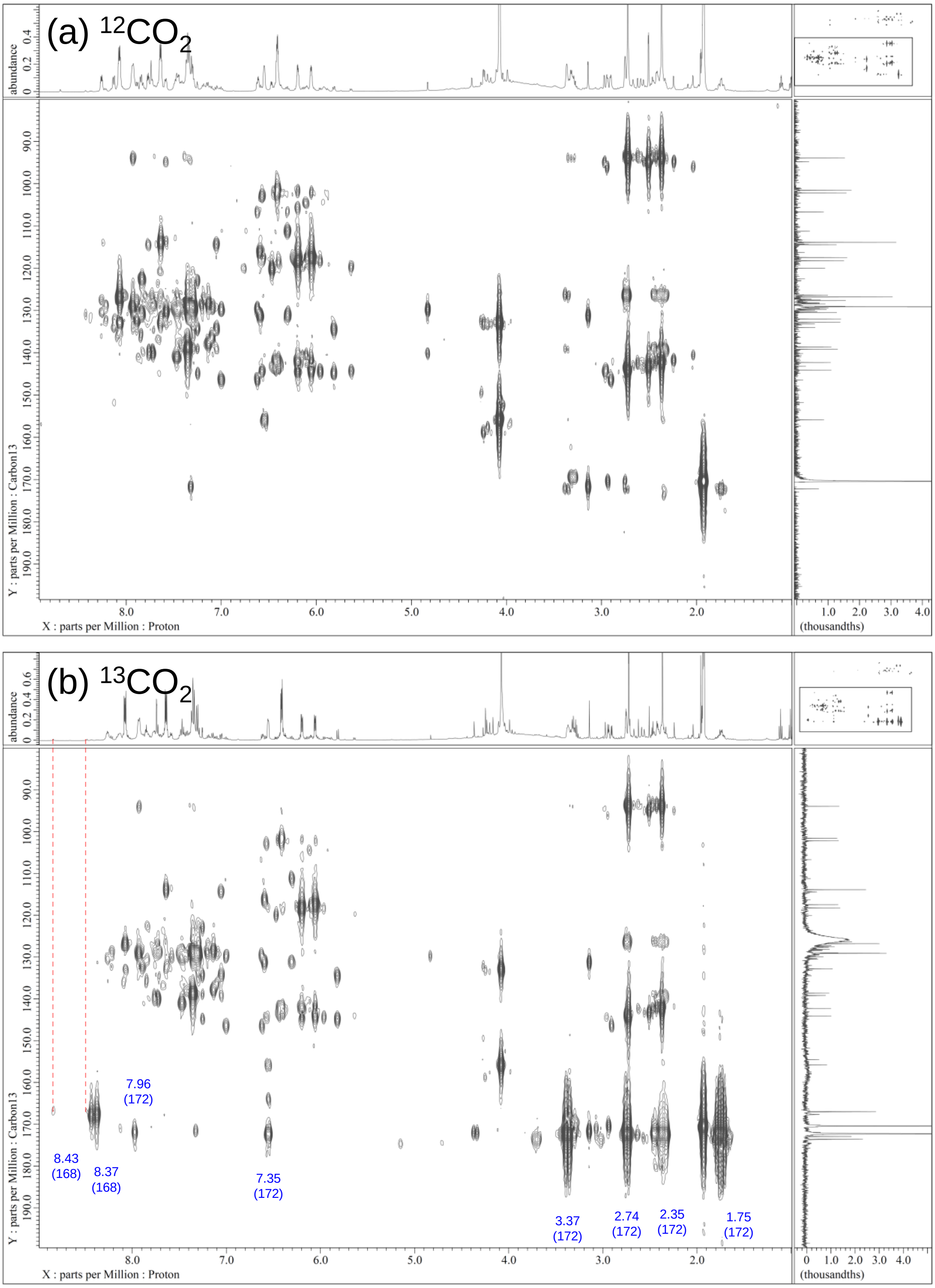

Figure S40. HMBC (DMA-*d*$_9$) of the reaction solutions containing **Fe$_2$-CP2$_p$** (100 μM), BIH (100 mM), and Phen2 (1 mM) during irradiation for 21 h at 420 nm (5 mW) under (a) $^{12}CO_2$ and (b) $^{13}CO_2$ atmospheres.

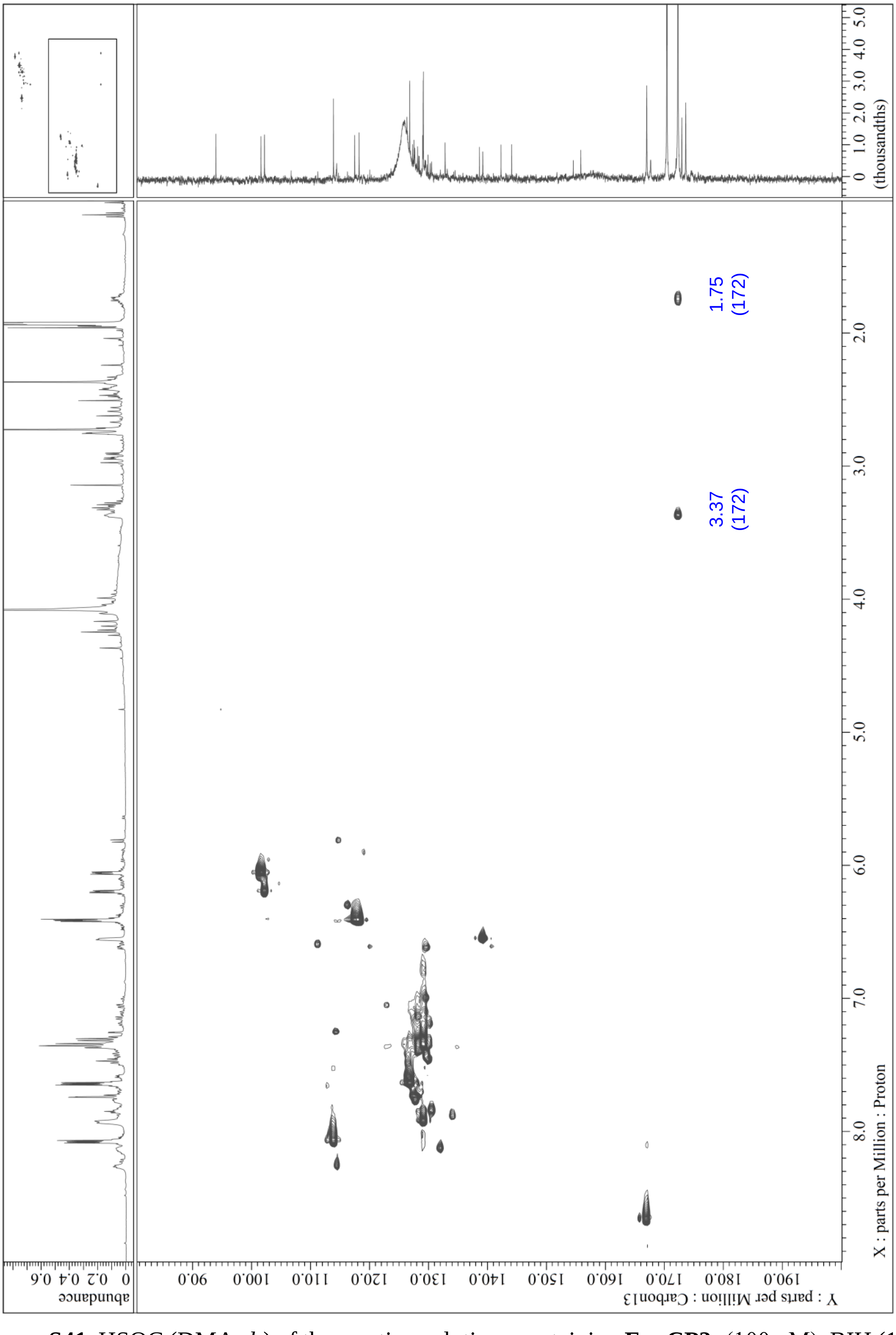

Figure S41. HSQC (DMA-*d*$_9$) of the reaction solutions containing **Fe$_2$-CP2*$_p$*** (100 μM), BIH (100 mM), and Phen2 (1 mM) during irradiation for 21 h at 420 nm (5 mW) under a $^{13}CO_2$ atmosphere.

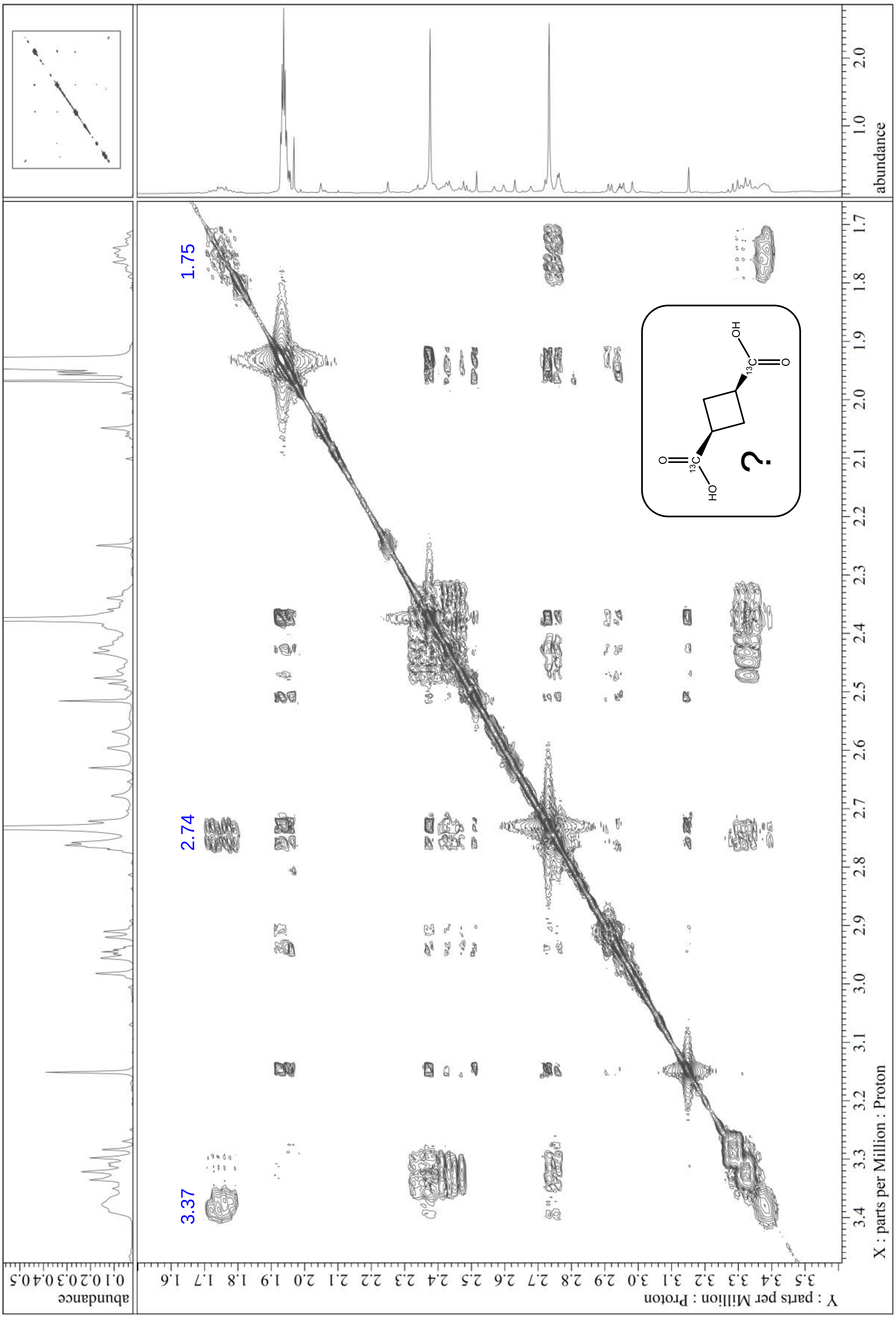

Figure S42. ^{1}H-^{1}H COSY (DMA-*d*$_9$) of the reaction solutions containing **Fe$_2$-CP2*$_p$*** (100 μM), BIH (100 mM), and Phen2 (1 mM) during irradiation for 21 h at 420 nm (5 mW) under a $^{13}CO_2$ atmosphere.
